# Development and testing of a multi-lingual Natural Language Processing-based deep learning system in 10 languages for COVID-19 pandemic crisis: A multi-center study

**DOI:** 10.3389/fpubh.2023.1063466

**Published:** 2023-02-13

**Authors:** Lily Wei Yun Yang, Wei Yan Ng, Xiaofeng Lei, Shaun Chern Yuan Tan, Zhaoran Wang, Ming Yan, Mohan Kashyap Pargi, Xiaoman Zhang, Jane Sujuan Lim, Dinesh Visva Gunasekeran, Franklin Chee Ping Tan, Chen Ee Lee, Khung Keong Yeo, Hiang Khoon Tan, Henry Sun Sien Ho, Benedict Wee Bor Tan, Tien Yin Wong, Kenneth Yung Chiang Kwek, Rick Siow Mong Goh, Yong Liu, Daniel Shu Wei Ting

**Affiliations:** ^1^Ministry of Health Holdings, Singapore, Singapore; ^2^Singapore National Eye Center, Singapore Eye Research Institute, Singapore, Singapore; ^3^Duke-National University of Singapore Medical School, National University of Singapore, Singapore, Singapore; ^4^Institute of High Performance Computing, Agency for Science, Technology and Research (A*STAR), Singapore, Singapore; ^5^Division of Digital Strategy Office, Singapore Health Services, Singapore, Singapore; ^6^Office of Innovation and Transformation, Singapore Health Services, Singapore, Singapore; ^7^Department of Head and Neck Surgery, Singapore General Hospital, Singapore, Singapore; ^8^Department of Urology, Singapore General Hospital, Singapore, Singapore; ^9^Tsinghua Medicine, Tsinghua University, Beijing, China

**Keywords:** Natural Language Processing, conversational chatbot, Artificial Intelligence, COVID-19, pandemic education, health education

## Abstract

**Purpose:**

The COVID-19 pandemic has drastically disrupted global healthcare systems. With the higher demand for healthcare and misinformation related to COVID-19, there is a need to explore alternative models to improve communication. Artificial Intelligence (AI) and Natural Language Processing (NLP) have emerged as promising solutions to improve healthcare delivery. Chatbots could fill a pivotal role in the dissemination and easy accessibility of accurate information in a pandemic. In this study, we developed a multi-lingual NLP-based AI chatbot, DR-COVID, which responds accurately to open-ended, COVID-19 related questions. This was used to facilitate pandemic education and healthcare delivery.

**Methods:**

First, we developed DR-COVID with an ensemble NLP model on the Telegram platform (https://t.me/drcovid_nlp_chatbot). Second, we evaluated various performance metrics. Third, we evaluated multi-lingual text-to-text translation to Chinese, Malay, Tamil, Filipino, Thai, Japanese, French, Spanish, and Portuguese. We utilized 2,728 training questions and 821 test questions in English. Primary outcome measurements were (A) overall and top 3 accuracies; (B) Area Under the Curve (AUC), precision, recall, and F1 score. Overall accuracy referred to a correct response for the top answer, whereas top 3 accuracy referred to an appropriate response for any one answer amongst the top 3 answers. AUC and its relevant matrices were obtained from the Receiver Operation Characteristics (ROC) curve. Secondary outcomes were (A) multi-lingual accuracy; (B) comparison to enterprise-grade chatbot systems. The sharing of training and testing datasets on an open-source platform will also contribute to existing data.

**Results:**

Our NLP model, utilizing the ensemble architecture, achieved overall and top 3 accuracies of 0.838 [95% confidence interval (CI): 0.826–0.851] and 0.922 [95% CI: 0.913–0.932] respectively. For overall and top 3 results, AUC scores of 0.917 [95% CI: 0.911–0.925] and 0.960 [95% CI: 0.955–0.964] were achieved respectively. We achieved multi-linguicism with nine non-English languages, with Portuguese performing the best overall at 0.900. Lastly, DR-COVID generated answers more accurately and quickly than other chatbots, within 1.12–2.15 s across three devices tested.

**Conclusion:**

DR-COVID is a clinically effective NLP-based conversational AI chatbot, and a promising solution for healthcare delivery in the pandemic era.

## 1. Introduction

The COVID-19 pandemic has profoundly changed our lives and disrupted global healthcare systems. The demand for medical services is increasing despite persistent movement and social contact limitations. This is further complicated by misinformation related to COVID-19 on the internet and social media (1, 2), which may thwart the implementation of public health measures. Healthcare institutions are therefore exploring alternative models to improve communication, diagnostics, and treatment (3), including the use of digital technology and big data to facilitate healthcare delivery and pandemic control (4). As such, platforms such as telemedicine, Artificial Intelligence (AI) and Natural Language Processing (NLP) chatbots have gained significant prominence (5).

Natural language remains a fundamental way information is communicated in the healthcare setting. NLP is a range of computational techniques used to automatically analyze and represent human language (6). It has multiple utilities including conversational chatbots, automated translation, smart assistants, and predictive text writing (7–9). With the capacity for “complex dialogue management and conversational flexibility,” AI applied in healthcare communication has the potential to benefit humans significantly (10). Chatbots could therefore fill a pivotal role in the dissemination and easy accessibility of accurate information in a pandemic, in an interactive manner akin to the conventional patient-physician communication. Voice chatbots are capable of automated acute care triaging, remote monitoring, and chronic disease management (11) NLP chatbots have also been useful in education, including radiation safety training for clinicians (12). Furthermore, chatbots have applications in oncology, including patient support, process efficiency, and health promotion (13).

COVID-19 related applications of NLP include computerized tomography reports analysis (14), as well as chatbots. Most COVID-19 chatbots currently are triaging tools or symptom checkers, whilst few are developed to answer open-ended questions (15). These include the World Health Organization (WHO) COVID-19 chatbot (https://www.facebook.com/WHO/), as well as the Centers for Disease Control and Prevention Coronavirus Self-Checker (https://www.cdc.gov/coronavirus/2019-ncov/symptoms-testing/coronavirus-self-checker.html) (16, 17). Healthcare workers have naturally been sought to answer open-ended queries regarding COVID-19, as they are viewed as dependable and trustworthy authority. The clinical need for general open-ended COVID-19 chatbots therein lies not only in reducing labor-intensive healthcare communication, but also serving as an accessible and reliable source of information for large-volume queries. A well-informed public would enable healthcare systems to reap benefits, including compliance with public health measures, and improved vaccination rates, amongst others.

Current medical COVID-19 chatbots face several limitations. First, most of these chatbots are created with English as the intended medium, thus limiting the utility for non-native English speakers (18). In comparison, there is a lack of viable multi-lingual chatbots (19). Next, achieving high accuracy may prove difficult due to nuances in communication. Inputs that are ambiguous or irrelevant to how the chatbot was trained can lead to a lack of meaningful responses by the chatbot (20). Our study aims to address these limitations by developing a multi-lingual chatbot able to respond accurately and quickly to general COVID-19 related questions by patients and the public.

## 2. Materials and methods

### 2.1. Chatbot overview

**Figure 1:**
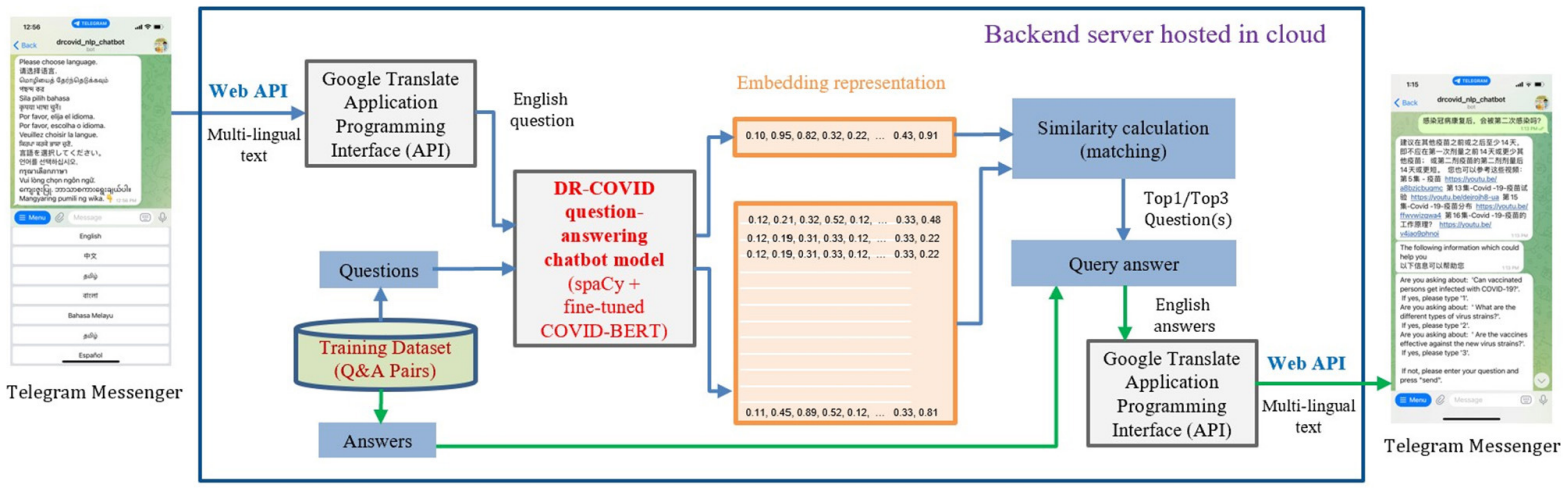
Overview of DR-COVID Natural Language Processing (NLP) chatbot usage and architecture.

In this multi-center prospective study, we developed a multi-lingual conversational chatbot, DR-COVID, hosted on the Telegram platform to answer COVID-19 related queries (Figure 1). The Telegram chatbot may be assessed at: https://t.me/drcovid_nlp_chatbot. English was used as the base to train the chatbot. A training dataset of unique questions-answer pairings was first created to train the NLP architecture, covering a range of common COVID-19 topics in the English language. Each question-answer pairing was expanded into sub-questions to increase the variety and scope of the training dataset. Subsequently, an independent testing dataset comprising questions-answer pairings in English was created to assess English accuracy. Collaborators were also involved in providing testing questions in nine widely-spoken non-English languages. These multi-lingual questions were translated to English questions using Google Translate Application Programming Interface (API). The questions in English were used as the input for our NLP ensemble model. Similarity calculation was used to retrieve the output, that is, the top 1 and top 3 closest matching answers. If the original question was not in English, then the output would be translated to English using Google Translate API, and subsequently displayed on the Telegram application. Our study did not involve patients, and was hence exempt from requirements of approval by the Singhealth Institutional Review Board.

### 2.2. Training and testing dataset

Inclusion criteria for input data were topics relating to COVID-19, whereas exclusion criteria were those not relevant to COVID-19. The training and testing question-answer pairs were developed sequentially in English, consolidating data from publicly available sources (Supplementary Table 1). The training dataset was developed by creating main unique questions paired with respective answer (MQA), and grouped based on WHO categories: general information, contact tracing, symptoms and treatment, personal protection, public health travel advisory, safe distancing, and vaccines (21). Poor quality or unavailable data were not included if unable to further improve upon, based on the available sources.

Two categories of MQA were created: Singapore-centric, and global. Singapore-centric questions were defined as those localized to Singapore geographically, specific to Singapore's population, policies, rules, and regulations. Global questions included those pertaining to global statistics, general information on COVID-19, and policies with impact on a global scale.

Each MQA was expanded into 5 to 15 unique sub-questions, and each sub-question grouped and identified for answer retrieval based on the corresponding MQA. Next, the training dataset was independently created with at least three questions per MQA. A total of 218 MQA pairings were developed from the period of 1^st^ Jan 2021 to 1^st^ Jan 2022. Data was vetted for repetition and grammar twice, and the finalized content vetted again.

The training dataset will be made available at GitHub on manuscript acceptance: https://github.com/leixiaofeng-astar/drcovid_nlp_chatbot.

### 2.3. Natural Language Processing chatbot architecture

**Figure 2:**
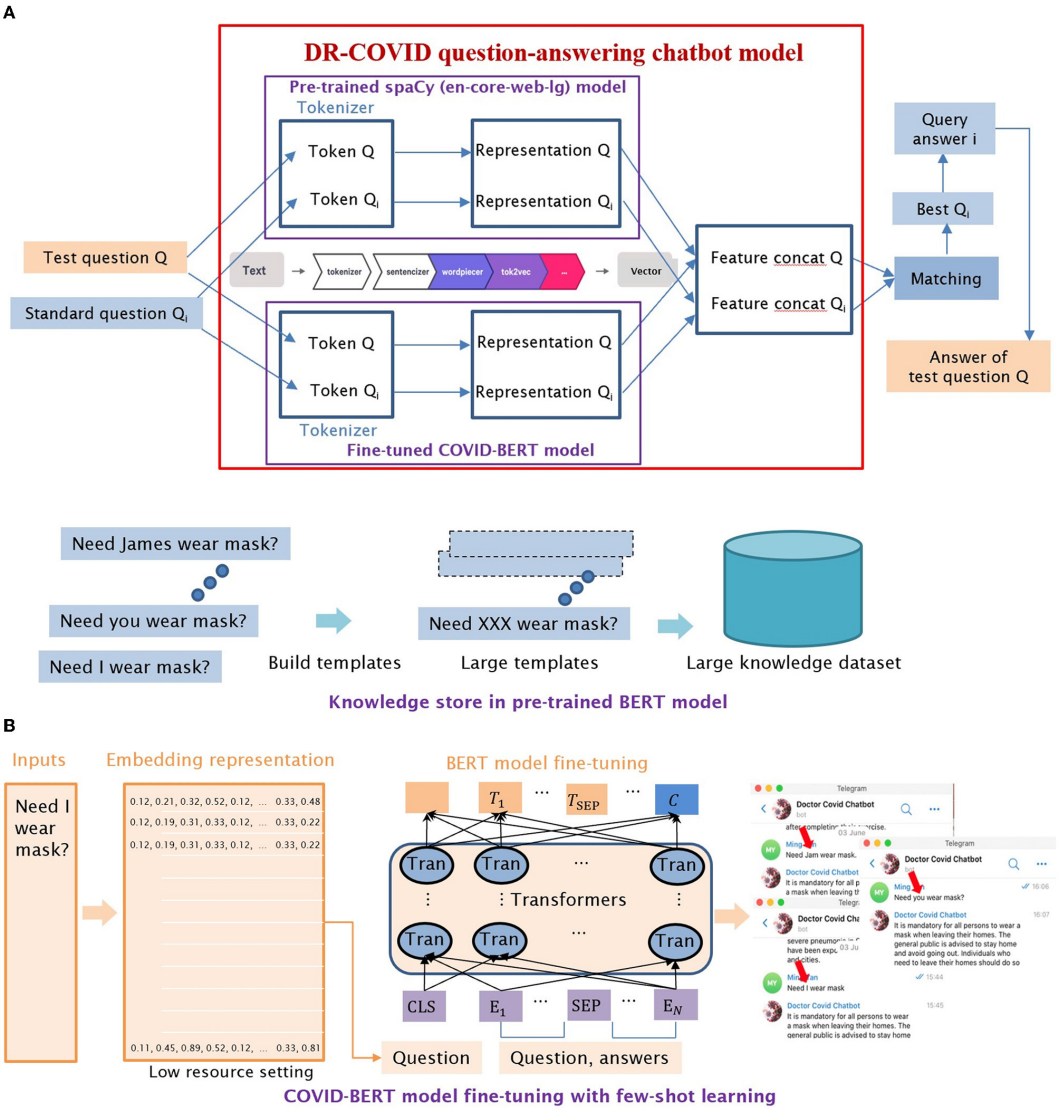
Detailed illustrations of DR-COVID Natural Language Processing (NLP) chatbot architecture. **(A)** Illustration of NLP ensemble model architecture, which combined the vectors of two models with different weights, with the new vector used for similarity calculation. **(B)** Illustration of few-shot learning, which enabled the customized BERT model to be better trained when a limited number of MQAs was available in the training dataset.

Two separate large corpus-trained NLP transformer models, spaCy library (22) and locally-modified Bidirectional Encoder Representation Transformer (BERT) (23), were combined in an 0.2: 0.8 weightage, to develop the chatbot architecture. The resultant vector was used for similarity calculation which was required for question-answer matching (Figure 2A). Few-shot learning, which involved task sampling instead of direct training using the input dataset, was utilized in a low-resource setting; this enabled our customized BERT model to be better trained when a limited number of MQAs was available in the training dataset (Figure 2B).

Rule-based question-answer retrieval was performed using feature extraction, and representation for the input test questions. Subsequently, a similarity score was generated for each MQA, with the highest matched score being the retrieved answer and therefore output. If similarity score fell below the pre-set threshold of 0.85 in our study, the top 3 closest matching MQAs were retrieved as the output instead.

### 2.4. Performance assessment

The ensemble model underwent three iterations of improvement before being used for eventual assessment. Chatbot performance was assessed based on the accuracy, AUC, precision, recall, and F1 score for the overall, and top 3 answers generated. A positive response was recorded for the top 3 answers if any one answer was appropriate. Grading was performed by two investigators independently. In the event of disparate grading, a discussion was held to reach a consensus, failing which a third investigator would provide the final decision. Subsequently, we invited ten collaborators to each contribute 20 English questions in an open-ended format, and thereafter assessed the performance of the new questions. Confidence intervals were calculated using R (v4.0.5).

### 2.5. Multi-lingual text translation

The NLP models were trained with English corpus. The backend software used Google Translate API to translate the target language question into English as an intermediary step language, followed by analysis and question-answer retrieval, and lastly re-translation by Google Translate API back to the target language. The selected target languages included Chinese, Malay, Tamil, Filipino, Thai, Japanese, French, Spanish, and Portuguese.

We invited collaborators to assess the multi-lingual aspect of DR-COVID, with each contributing 20 questions in an open-ended format to assess the accuracy of the generated response. Ten collaborators were invited to assess the chatbot in Chinese and Malay; two in Spanish; and one each for the remaining languages Tamil, Filipino, Thai, Japanese, French, and Portuguese.

### 2.6. User interface assessment

Twenty questions with no overlap of MQA were selected for User Interface (UI) assessment (Supplementary Table 2), which involved timing the interval between question input and answer generation for DR-COVID, WHO Messenger (16) and National Health Service (NHS) Inform (https://ask.nhsinform.scot/) (24). This was performed on 1^st^ Feb 2022 by a single investigator, using a stopwatch on three digital devices, including laptop, tablet, and smartphone. Specifications are described in **Table 3**.

### 2.7. GPU vs CPU assessment

The deployments of DR-COVID chatbot application were compared, to highlight the differences in the throughput performance of Graphical Processing Units (GPU) vs. Central Processing Units (CPU). In this study, benchmarking tests were performed between CPU and GPU. NVIDIA TITAN Xp GPU and Intel(R) Xeon(R) W-2145 CPU were used during the evaluation. Data regarding memory usage with sequential time profiler and memory profiler was obtained using 100 users and 3 questions.

## 3. Results

A total of 2,728 questions in English, comprising 12,90 Singapore-centric and 1,438 global questions, were developed for the training dataset. Eight hundred twenty-one new questions in English were created as the testing dataset for assessment of accuracy, consisting of 335 Singapore-centric and 486 global questions (Supplementary Table 3).

### 3.1. Performance assessment

In terms of primary outcomes of interest, DR-COVID achieved an overall accuracy of 0.838 [95% confidence interval (CI): 0.826–0.851], comprising a proportion of 0.812 [95% CI: 0.791–0.832] correct Singapore-centric answers, and 0.856 [95% CI: 0.841–0.871] correct global answers. The top 3 accuracy was 0.922 [95% CI: 0.913–0.932], comprising 0.895 [95% CI: 0.881–0.914] and 0.940 [95% CI: 0.931–0.951] correct Singapore-centric and global answers, respectively (Table 1).

**Table 1:** Performance assessment for DR-COVID question-answer retrieval for overall and top 3 results, across both Singapore-centric and global questions.

**Mean [95% CI]**	**Overall**			**Top 3**		
	**Overall**	**Singapore-centric**	**Global**	**Overall**	**Singapore-centric**	**Global**
Accuracy	0.838 [0.826–0.851]	0.812 [0.791–0.832]	0.856 [0.841–0.871]	0.922 [0.913–0.932]	0.895 [0.881–0.914]	0.940 [0.931–0.951]
AUC	0.917 [0.911–0.925]			0.960 [0.955–0.964]		
Precision	0.864 [0.852–0.876]			0.938 [0.931–0.946]		
Recall	0.835 [0.822–0.850]			0.920 [0.910–0.929]		
F1 score	0.829 [0.818–0.841]			0.918 [0.911–0.925]		

AUC, area under the receiver operating characteristic curve; CI, confidence interval. Parameters are as follows–Threshold similarity score was set at 0.85. DR-COVID was trained on two Nvidia Titan RTX (24GB each) graphics processing unit (GPU), executed on either Nvidia Titan RTX (24GB) GPU machine or central processing unit (CPU) machine (64GB), and hosted on Telegram.

**Figure 3:**
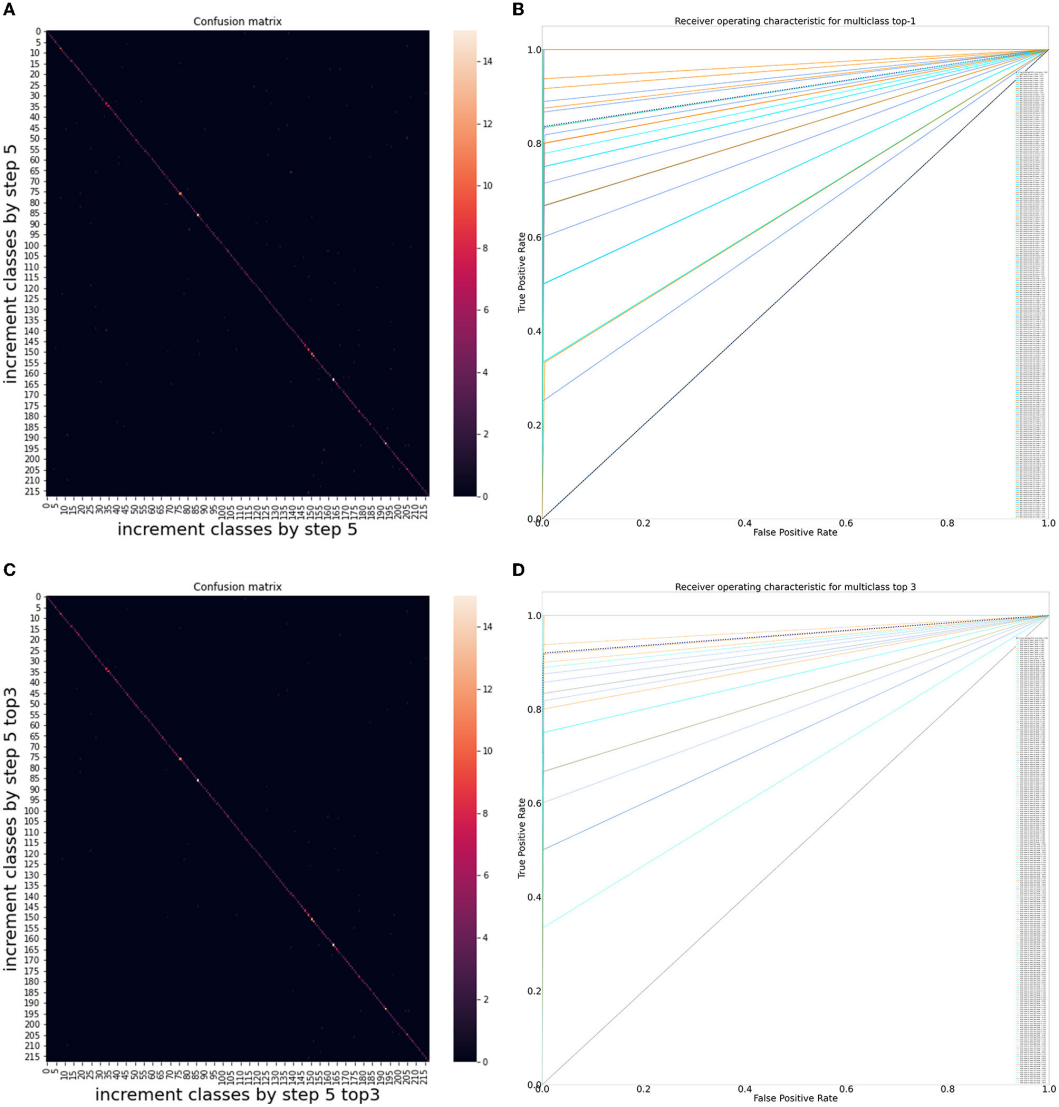
Performance of DR-COVID, our multi-lingual Natural Language Processing (NLP)-based deep learning system. **(A)** Confusion matrix diagram of algorithm predictions for overall results. **(B)** Receiver Operating Characteristic (ROC) curve for overall results. The dotted black line shows the average performance of the system in achieving the correct corresponding standard answer, with an area under the ROC curve (AUC) of 0.917 [95% confidence interval (CI): 0.911–0.925], precision of 0.864 ± 0.193 [95% CI: 0.852–0.876], recall of 0.835 ± 0.218 [95% CI: 0.822–0.850], and F1 score of 0.829 [95% CI: 0.818–0.841]. **(C)** Confusion matrix diagram of algorithm predictions for top 3 results. **(D)** Receiver Operating Characteristic (ROC) curve for top 3 results. The dotted black line shows the average performance of the system in achieving the correct corresponding standard answer, with an area under the ROC curve (AUC) of 0.960 [95% CI: 0.955–0.964], precision of 0.938 ± 0.124 [95% CI: 0.931–0.946], recall of 0.920 ± 0.145 [95% CI: 0.910–0.929], and F1 score of 0.918 [95% CI: 0.911–0.925].

Chatbot performance assessment can be formulated as a classification problem, and its accuracy validated in qualitative and quantitative manners, where each MQA belongs to a particular class. Receiver Operating Characteristic (ROC) analysis was performed to assess the classification problem, which enable values of AUC, precision, recall, and F1 score to be gleaned. For overall results, the AUC was 0.917 [95% CI: 0.911–0.925], precision was 0.864 ± 0.193 [95% CI: 0.852–0.876], recall was 0.835 ± 0.218 [95% CI: 0.822–0.850], and F1 score was 0.829 [95% CI: 0.818–0.841]. For top 3 results, all metrics showed an improvement; AUC was 0.960 [95% CI: 0.955–0.964], precision was 0.938 ± 0.124 [95% CI: 0.931–0.946], recall was 0.920 ± 0.145 [95% CI: 0.910–0.929], and F1 score was 0.918 [95% CI: 0.911–0.925]. The confusion matrix diagram and ROC curve for overall results and top 3 results are shown in Figures 3A–D, whereas the confusion matrix table is available on GitHub: https://github.com/leixiaofeng-astar/drcovid_nlp_chatbot. Greater details of ROC calculation and its parameters have been appended in Supplementary Text 1.

Ten collaborators were invited to each contribute 20 questions in the English language (Supplementary Table 4). When tested on the 200 new questions, DR-COVID achieved a decline in the overall accuracy to 0.550 [95% CI: 0.519–0.588], and top 3 accuracy to 0.660 [95% CI: 0.625–0.694].

### 3.2. Multi-lingual performance

In terms of secondary outcomes of interest, nine non-English languages were assessed for accuracy, using a total of 560 questions contributed by the collaborators (Supplementary Table 5). Supplementary Figure 1 and Supplementary Video 1 demonstrate the chatbot interface and response to an example question, “what are the available vaccines?”, in the selected languages. Portuguese performed the best overall at 0.900, followed by Spanish at 0.725, then Thai at 0.600 (Table 2).

**Table 2:** Multi-linguicism assessment for DR-COVID showing the top 3 performing non-English languages.

**Language**	***N***	**Overall accuracy**	**Top 3 accuracy**
Thai	20	0.600	0.700
Spanish	40	0.725	0.800
Portuguese	20	0.900	0.900

### 3.3. User interface assessment

In terms of other secondary outcome measures, DR-COVID achieved the highest overall accuracy of 0.800 when tested on the selected global questions, followed by WHO Messenger at 0.600, and finally NHS Inform at 0.500. The average time taken for DR-COVID to generate an answer was 2.15s ± 0.31 on a laptop device, 1.26 ± 0.49 on a tablet, and 1.12s ± 0.44 on a phone; significantly faster compared to NHS Inform, and WHO Messenger across all digital devices (*p* < 0.05). Amongst other question-answer chatbots tested for comparison, the average time taken for NHS Inform ranged between 2.20 and 2.51s, whereas that of WHO Messenger ranged between 4.04 and 4.85s (Table 3).

**Table 3:** Accuracy and user interface assessment for DR-COVID and other enterprise-grade chatbot systems, across three digital devices.

**Question-answer chatbot**	**Accuracy**	**Mean time taken per question (s)** ± **SD**		
	**Best answer**	**Laptop**	**Tablet**	**Smartphone**
DR-COVID (GPU)	0.800	2.15 ± 0.31	1.26 ± 0.49	1.12 ± 0.44
WHO messenger	0.600	4.63 ± 0.88 (*p* < 0.01)	4.04 ± 1.21 (*p* < 0.01)	4.85 ± 0.65 (*p* < 0.01)
NHS inform	0.500	2.43 ± 0.43 (*p* = 0.03)	2.51 ± 0.69 (*p* < 0.01)	2.20 ± 0.46 (*p* < 0.01)

SD, standard deviation; p, p-value at significance level of 0.05, using a two-tailed t-test, in comparison to DR-COVID for the corresponding device; GPU, graphics processing unit; WHO, World Health Organization; NHS, National Health Service. Device specifications are as follows–Laptop: Microsoft Surface Pro 6, Intel(R) Core(TM) i5-8250U CPU @ 1.60GHz, 1.80 GHz, 256GB memory. Tablet: iPadMini 4, iOS 14.8.1, 128GB memory. Smartphone: iPhone 11, iOS 14.7.1, 128 GB memory.

### 3.4. GPU vs CPU assessment

The tests were conducted by running a DR-COVID chatbot application on a workstation with Ubuntu 16.04.7 LTS operating system, NVIDIA TITAN Xp 12GB GPU RAM and Intel(R) Xeon(R) W-2145 CPU @ 3.70GHz 64 GB DDR4 RAM. Using 100 users and three questions each as the test condition, and the sequential time and memory usage recorded was the average throughput 20 times test. To run DR-COVID chatbot on CPU required 3.628 GB, whereas on GPU required 2.936 GB memory plus 600 MB CPU memory. The average time taken per question was 23.52 s for CPU, whereas in comparison, the GPU could achieve a 66.9% reduction in time, at 7.79 s.

## 4. Discussion

In this multi-center study, we created a general question-answer chatbot with a training dataset of 2,728 questions to address COVID-related enquiries, incorporated multi-lingual text to text translation, and assessed chatbot performance by evaluating accuracy through external open-ended assessments, and comparing technical performance against enterprise-grade chatbot systems. In terms of primary outcomes of interest, we achieved overall and top 3 accuracies of 0.838 [95% confidence interval (CI): 0.826–0.851] and 0.922 [95% CI: 0.913–0.932] respectively. For overall results, the AUC was 0.917 [95% CI: 0.911–0.925], precision was 0.864 ± 0.193 [95% CI: 0.852–0.876], recall was 0.835 ± 0.218 [95% CI: 0.822–0.850], and F1 score was 0.829 [95% CI: 0.818–0.841]. For top 3 results, the AUC was 0.960 [95% CI: 0.955–0.964], precision was 0.938 ± 0.124 [95% CI: 0.931–0.946], recall was 0.920 ± 0.145 [95% CI: 0.910–0.929], and F1 score was 0.918 [95% CI: 0.911–0.925]. In terms of secondary outcomes of interest, we demonstrated multi-linguicism with 9 non-English languages using an external open-ended testing approach, as well as higher speed and accuracy compared to other chatbots.

In our study, we used an ensemble model aiming to overcome technical challenges associated with a single architecture, which gives lower accuracy, higher variance, noise, and bias (25). The ensemble method reduces model error while still preserving its generalization (26). In addition, accuracy and diversity can be improved by optimizing the performance of each base estimator and incorporating a range of estimators respectively. Ensemble models have typically outperformed single classifiers in terms of AUC, accuracy, and recall (27, 28). Considerations for implementation should include risks of overfitting the training data, as well as costs and complexity of training and deployment.

Existing literature regarding NLP-based chatbots in the COVID-19 pandemic has been largely experimental or descriptive in nature (29, 30). Nonetheless, studies thus far have demonstrated accuracies ranging between 0.54 and 0.92 (31–33). A Canadian chatbot, Chloe, developed to address pandemic misinformation, has demonstrated accuracies of 0.818 and 0.713 for the English and French language respectively, using a BERT-based NLP architecture (31). Whilst we demonstrated a better overall accuracy of 0.838 in the English language–potentially contributed by our ensemble vs. single classifier model–our accuracy of 0.350 in the French language fell short of expectations. There were several factors that could account for this discrepancy. First, Chloe was developed in the context of a bilingual English and French-speaking populace. Questions in the French language were able to undergo direct question-answer retrieval, without the use of translation software. On the contrary, DR-COVID required the use of Google Translate as an intermediary step, before question-answer retrieval, as well as before providing the output in the French language. Google Translate is not capable of transcreation, that is, the correct interpretation of context, intent, cultural and language nuances (34). As a result, non-native translation such as in DR-COVID, is ultimately less ideal than native translation, due to contextual specificities and transcreation difficulties. It may also be of utility for other chatbots to share their questions tested, in order to draw a reasonable comparison. Potential solutions would include collaborating with international partners and native translators to fine tune the multi-lingual datasets, as well as align with the locale, with the understanding that this would entail necessary cost, retraining, and turnaround time. In particular, Singapore is intrinsically a multi-racial and multi-lingual society, with a significant international populace. As such, it will be worthy to invest these resources, and shall be to the strength that we can produce such a chatbot as well.

Next, there were several questions in French of a highly specific nature, which were not within the scope of our existing MQAs, including “can I get infected through aerated steam?” (Supplementary Video 2). Furthermore, answers to questions such as “can I get delta after being in remission from alpha?” were not included in our original dataset, as data regarding reinfection with new variants was not available at the time of development. That said, while it is a fair point that highly specific or technical questions may be difficult to achieve accuracy on initial try, more common layman queries that appear in every language should minimally be answered. In this study, multi-lingual analysis was limited by the small number of testing questions with *N* = 20 on average, lest the Chinese and Malay languages. Nevertheless, an ongoing analysis is underway to garner and assess more questions for multi-lingual accuracy, as well as to evaluate if the differential accuracy may be attributed to technical or general questions.

Another Tunisian chatbot Smart Ubiquitous Chatbot, based on Long Short-Term Memory (LSTM) networks, was developed for education, and stress management during the pandemic. It reported an accuracy of 0.92, precision of 0.866, recall of 0.757, and F1 score of 0.808 (32). Similarly, DR-COVID achieved precision of 0.864 comparable to Smart Ubiquitous Chatbot, but demonstrated higher recall of 0.835, that is, the capability of giving more of the correct answers amongst all the correct answers. We also achieved a higher F1 score of 0.829, meaning that taking precision and recall in tandem, our chatbot demonstrated better overall performance. Extrinsic differences in linguistics, local policies and populations, as well as intrinsic technicalities of the algorithms likely play a role in these differential results. We were however unable to compare top 3 accuracy, recall, and precision with other chatbots that lacked this function. There was also difficulty benchmarking our AUC against other COVID-19 chatbots, as there has been a paucity of research evaluating this metric thus far.

We demonstrated that when tested on new questions in English provided by collaborators, DR-COVID fared less optimally, with a drop in accuracy from 0.838 to 0.550, compared to using our own testing dataset. These errors are perhaps explainable. Firstly, this variance may illustrate the differential perspectives between the medical community and general public. The training and testing datasets, developed by the internal team comprising medical practitioners and data scientists, tend to be more medical in nature, including “will the use of immunomodulators be able to treat COVID-19?”, and “what is the mechanism of action of rapid COVID-19 tests?”; there was potentially selection bias to some degree. On the other hand, the external questions were contributed by collaborators of both medical and non-medical backgrounds; these relate more to effects on daily life, and coping mechanisms. For example, “is the hospital safe to visit?”. This further illustrates the limitations in our training dataset in covering everyday layman concerns relating to COVID-19 as discussed previously, and therefore potential areas for expansion. That said, we do observe common topics of overlap, such as general information, symptoms, and treatment pertaining to COVID-19.

Secondly, despite having undergone several cycles of retraining, our model might not have the most up-to-date information on certain questions. Chatbots require a tedious training and retraining process. Information and policies are constantly changing in a pandemic setting, on both a local and global scale, which necessitates frequent monitoring and updating of the model, to ensure that appropriate information is conveyed. A prime example would be vaccine-related information such as booster dose requirements, newly approved vaccines, and variant-specific efficacy. Our model was not equipped with new information regarding booster vaccines, and was therefore shorthanded in addressing these questions. To circumvent tedious retraining, we could consider reinforcement learning in future implementation, a technique which incentivises the chatbot to learn through trial and error, by “rewarding” correct outputs and “punishing” incorrect answers (35).

Thirdly, insofar as our knowledge regarding COVID-19 is constantly evolving, there remain uncertainties for which it is challenging to give definite answers to. Questions such as “when will the COVID-19 pandemic end?” are difficult to predict, may give seemingly unsatisfactory answers, and therefore affect the accuracy of the chatbot. Ultimately, this difference demonstrates the variability which may arise, and therefore the need to test chatbots externally when implemented in a real-world setting.

This study gives hope to the potential expansion and real-world implementation of NLP-DLS chatbots, such as DR-COVID. The use of open-source translation software, with the caveat of its drawbacks as discussed earlier, may improve scalability and multi-lingual customizability. Moreover, integration onto social media platforms–such as Telegram in our case–enables greater reach and convenience, potentially removing geographical constraints (2); the WHO's global pandemic outreach through WhatsApp is a prime example (36). These could mitigate resource limitations by improving scalability and efficiency (37). Moreover, chatbots have a high handling capacity which allows simultaneous conversations with multiple users (38), and are instantly available on-demand. This provides patients with a reliable source of information, whilst helping off-load labor-intensive communication traditionally performed by healthcare workers.

Furthermore, information garnered from multiple reliable sources can be presented in a succinct manner, mitigating the dangers of online misinformation (39). Specific to the ongoing pandemic, DR-COVID and other NLP chatbots could fill a pivotal role in the dissemination and easy availability of accurate information regarding COVID-19, therein also facilitating implementation of pandemic measures. They could potentially serve as accessible platforms to disseminate new operational workflow, news and protocols, thereby minimizing confusion faced on the ground by the general population, and even healthcare workers. This is critical to manage large-volume queries and national measures, which are often challenging and require unparalleled effort to coordinate on a large-scale. Moreover, this matters because misinformation could translate to vaccine hesitancy, and reluctance to comply with public health measures such as mask-wearing. On the other hand, a better understanding of COVID-19 would reduce panic amongst the public, thereby reducing unwarranted visits to the emergency department, and better optimizing resource allocation in healthcare systems. Moreover, the resultant higher vaccination rates would also enhance “herd immunity,” thereby reducing the transmission of COVID-19 with resultant mortality benefits.

Lastly, whilst the main purpose of DR-COVID has been to facilitate efficient and accurate information sharing, it may be of utility to explore the inclusion of other tools, including detection of misleading information, triage, risk assessment, monitoring, and general wellbeing. For example, both ensemble- and BERT based DL systems have demonstrated utility in detecting COVID-19 related misinformation on the internet and social media (40, 41). Another COVID-19 chatbot developed by the University of Pennsylvania Health System included a symptom checker for self-triaging (30), whereas Ana, a Brazilian chatbot, guided users regarding the indications for seeking inpatient treatment (42). These can assist in triaging patients to suitable echelons of care, and thereby potentially reduce unwarranted health-seeking behavior. That said, one has to bear in mind the caveat that AI would not grasp the nuances of clinical management, and that liability issues for triaging errors should be addressed before implementation. In terms of risk stratification, another chatbot developed by University of California, San Francisco Health, assisted the hospital in making real-time manpower decisions, based on exposure risk of its healthcare workers (43). Another Singaporean chatbot, Bot MD, has helped doctors prioritize attention to potentially unwell patients on COVID-19 home recovery (44). Finally, chatbots have also been used to monitor the psychological effects, and mitigate the implications of isolation caused by social distancing (45).

COVID-19 is likely to become endemic in time to come (46). In envisioning the eventual implementation for the current pandemic and beyond, we are also cognisant regarding the importance of acceptability and useability, which should be optimized for real-world implementation (47); in fact, the primary factor influencing acceptability is perception of ability, which is in turn driven by trust in the system (48). When implemented in the real world, there is therefore a need to balance between presenting facts from global authorities such as the WHO, and vocalizing local perspectives and policies. This requires collaboration amongst stakeholders. Therein also raises questions regarding legislative responsibility and accountability for chatbots. Decisions regarding licensing, much like credentials for healthcare workers, would require further deliberation.

Planned future studies include expansion to more languages, and integration to web messenger and social media platforms to reach greater audiences. Conversational experience can be refined with contextual awareness to improve relevance of answer retrieval. Future directions would also entail exploration of different but complementary domains such as text-to-speech, and speech-to-speech, which may be of help in specific populations like the visually impaired, or to provide more options for convenience. Other potential use cases in pandemic management include NLP-based risk stratification, contact tracing, and patient monitoring. Finally, DL-based chatbots may be utilized in various medical and surgical specialities for targeted patient education, disease monitoring, and encouraging treatment compliance, amongst others.

## 5. Conclusion

Chatbots utilizing NLP, a type of conversational AI, have emerged as promising solutions to improve healthcare delivery in the pandemic era. In this study, we developed a multi-lingual NLP-based AI chatbot, DR-COVID, to facilitate healthcare delivery and disease control. Our NLP model, utilizing the ensemble architecture, achieved overall and top 3 accuracies of 0.838 [95% CI: 0.826–0.851], and 0.922 [95% CI: 0.913–0.932] respectively. For overall and top 3 results, AUC scores of 0.917 [95% CI: 0.911–0.925] and 0.960 [95% CI: 0.955–0.964] were achieved respectively. The sharing of training and testing datasets on an open-source platform will also contribute to existing data. Whilst AI-based NLP chatbots can enable healthcare systems to reap public health and resource benefits, clinicians and policymakers should work in tandem to deliver solutions to potential problems in real-world implementation.

## Data availability statement

The original contributions presented in the study are included in the article/Supplementary material, further inquiries can be directed to the corresponding author.

## Author contributions

LY and WN created the training and testing dataset, collected data, and contributed to study conceptualization. ST and ZW contributed to creation of the training and testing dataset. XL, MY, MP, and XZ conceptualized the methodology of the chatbot model, trained the chatbot, and performed the statistical analysis. LY wrote the first draft of the manuscript. DT provided the overall leadership, conceptualized the study, and as well as procured funding. All authors contributed to manuscript revision and approved the submitted version.
