# Supplementary material for: Development and testing of a multi-lingual Natural Language Processing-based deep learning system in 10 languages for COVID-19 pandemic crisis: A multi-center study
Source: Front Public Health. 2023 Feb 13;11:1063466. doi: 10.3389/fpubh.2023.1063466 (PMC9968846; doi:10.3389/fpubh.2023.1063466)
Supplement: Supplementary file 1 [file Data_Sheet_1.docx]

**Supplementary Information**

**Supplementary Figure 1.** Demonstration of chatbot interface and response to “what are the available vaccines?” in ten languages. **A)** English, **B)** Chinese, **C)** Malay, **D)** Tamil, **E)** Filipino, **F)** Thai, **G)** Japanese, **H)** French, **I)** Spanish, and **J)** Portuguese.

**
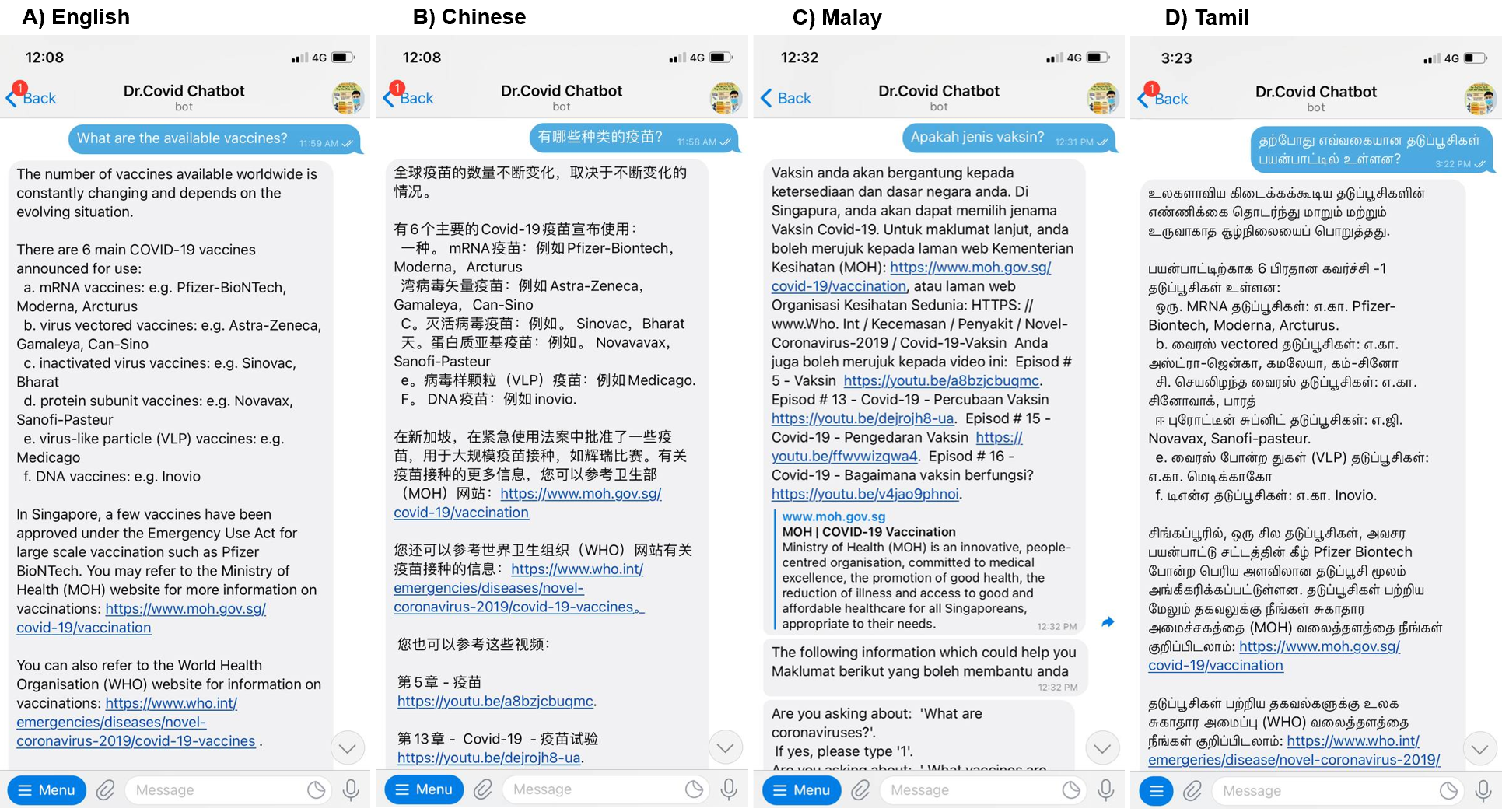
**


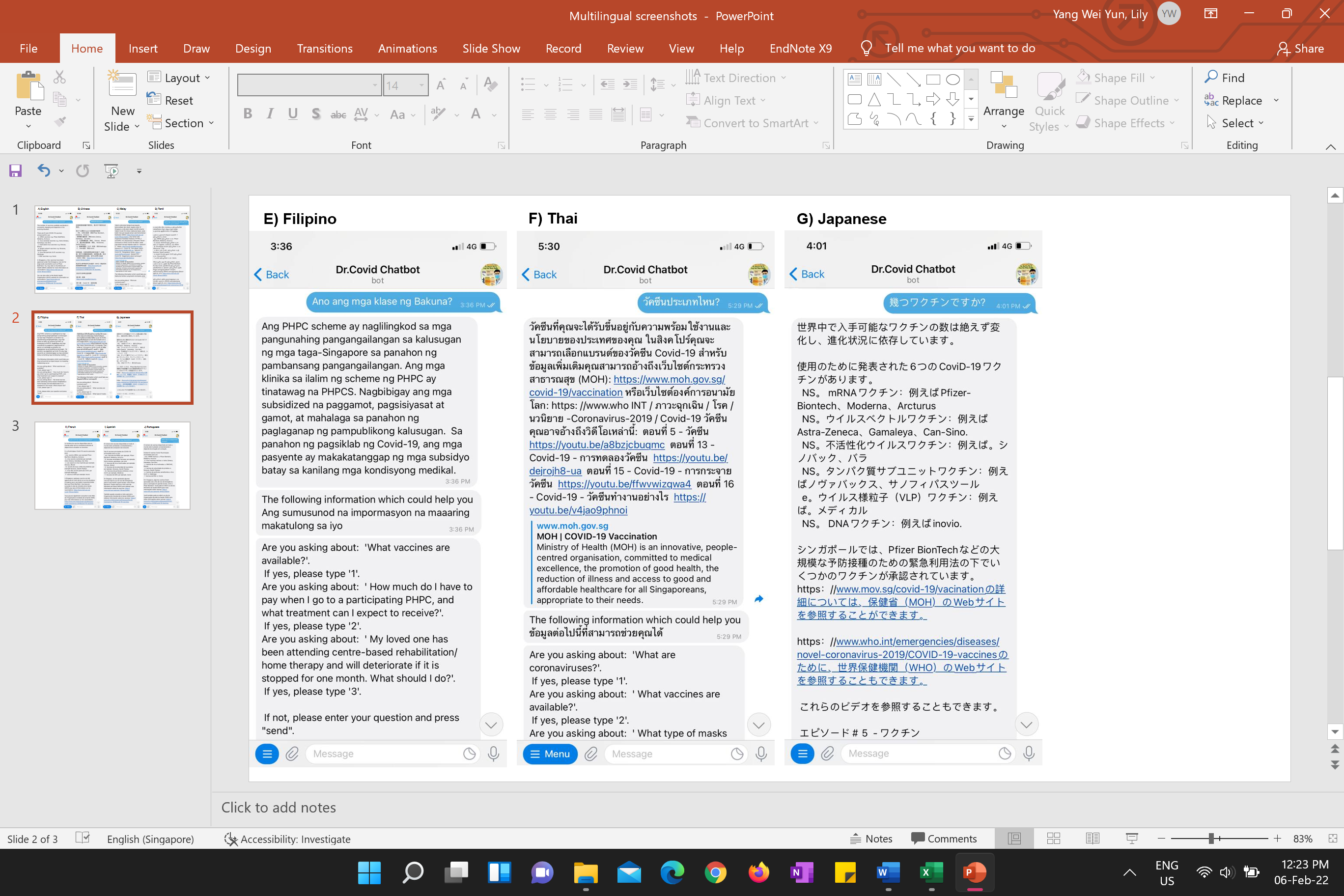


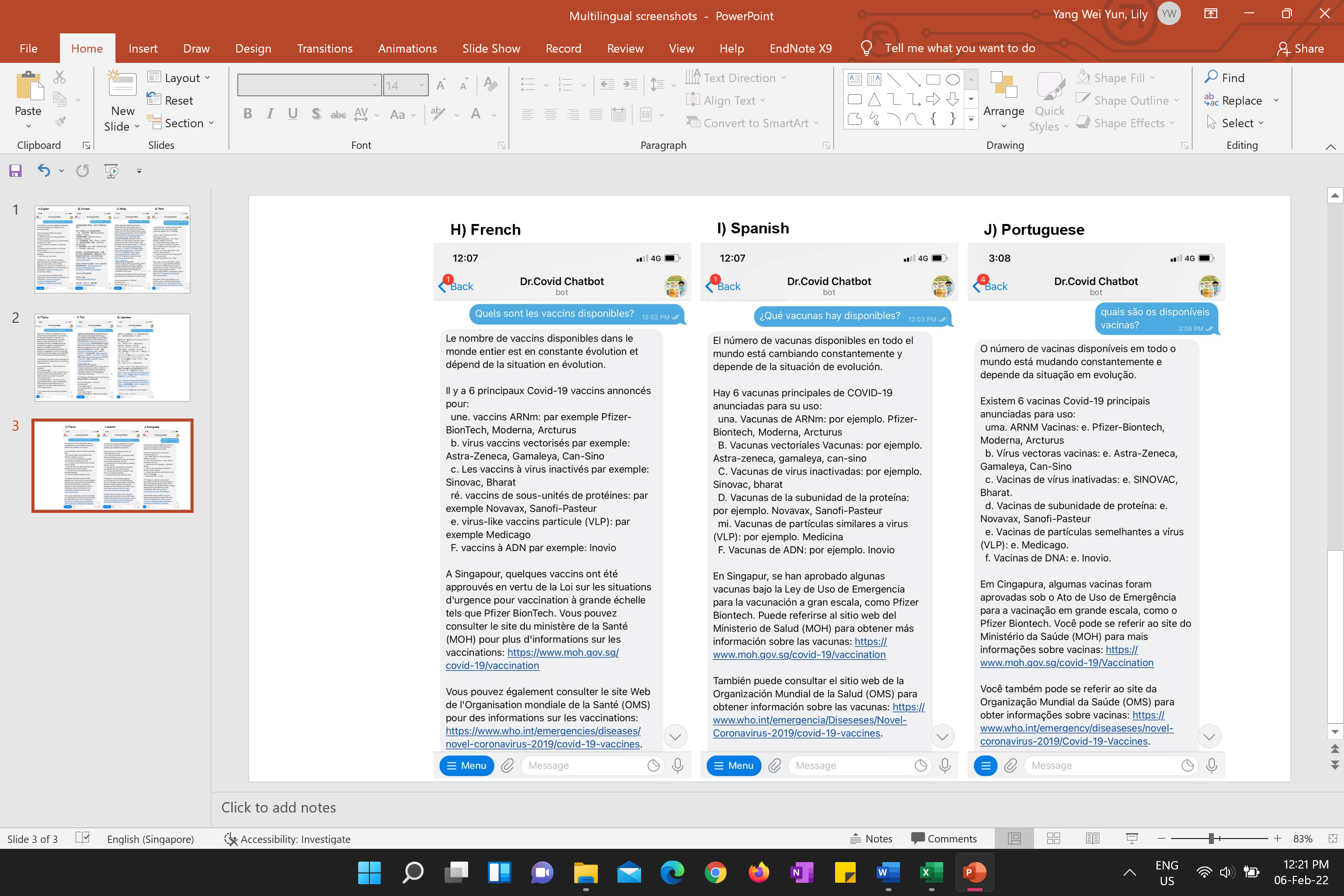


**Supplementary Text 1.** Detailed explanation of Receiver Operating Characteristic (ROC) calculation and its parameters.

The Receiver Operating Characteristic (ROC) is a graph that maps the relationship between the true positive rate (TPR) and the false positive rate (FPR). The true positive rate is the proportion of observations that are correctly predicted to be positive out of all positive observations (TP/ (TP + FN)). Similarly, the false positive rate is the proportion of observations that are incorrectly predicted to be positive out of all negative observations (FP/ (TN + FP)).

We formulate chatbot performance assessment as a classification problem. The test dataset consists of 821 questions and 218 answers. Each answer represents one class. Hence, there are 218 classes.

On average, we have 2 to 5 different types of questions per class (2 to 5 variants of questions that provide the same answer). As an example, a particular class is defined as follows:

*a) How long can COVID-19 remain on surfaces?*

*b) Can I get COVID-19 by shaking someone's hand?*

*c) How does the spreading of COVID-19 take place?*

The response answer to all these queries is:

*COVID-19 is transmitted primarily through respiratory droplets. The virus is carried within droplets from an infected person over a short distance from coughing and sneezing. These droplets can infect someone else through the eyes, nose or mouth, directly or indirectly through hands. You are advised to remain vigilant and observe good personal hygiene practices. Besides, respiratory droplets from an infected person can land on objects and surfaces. Someone can become infected by touching a contaminated surface, object or the hand of an infected person and then touching their own mouth, nose or eyes. Current data shows that the virus can live for at least 72 hours on plastic and stainless steel, up to 4 hours on copper, and up to 24 hours on cardboard. However, the data was collected under laboratory conditions and should be interpreted carefully.*

*Transient contact with an infected person is low. Examples of transient contact include passing by on public transport. But the longer you are in contact, the greater the risk.*

*In Singapore, MOH is closely monitoring the evolving situation and members of public are advised to check the MOH website for updates. https://www.moh.gov.sg/covid-19*

Three different categories of questions relate to the method of spread, and lead to the same answer for each of these questions. Hence, the answer would be the same for all these questions, so they belong to a particular class.

In the multi-class setting, we can visualize the performance of multi-class models according to their one-vs-all precision-recall curves. The area under the ROC curve (AUC) can also be generalized to the multi-class setting.

We pass all the questions in the test dataset through our natural language processing (NLP) model to identify corresponding similar questions and fetch the answer corresponding to the query. Here **true labels** are our questions from the test dataset and **predicted labels** are the question response obtained by passing the test dataset question to the NLP model. For each class, we compute the following:

a) **True Positive (TP)**: Test dataset question is passed to NLP model, and the most similar response question obtained from the model belongs to the same class.

b) **False Negative (FN)**: Test dataset question is passed to NLP model, but the most similar response question obtained from the model belongs to a different class.

c) **False Positive (FP)**: Test dataset question is passed to NLP model, but the model labels it as a different test question.

d) **True Negative (TN)**: The NLP model correctly labels a false test question as belonging to a wrong class.

Finally, we compute the TPR and FPR rates using the formulae

TPR = (TP/ (TP + FN))

FPR = (FP/ (TN + FP))

The ROC curve is then computed by plotting TPR vs FPR. We compute the AUC score for each class. The final performance measure is the AUC score for each class. The AUC shown in results section is the average AUC of all 218 classes.

**Supplementary Video 1.** Demonstration of chatbot interface and response to “what are the available vaccines?” in ten languages.

1. English: <https://youtu.be/mCMt5fVDXdQ>
2. Chinese: <https://youtu.be/vqmB_Zsv6qY>
3. Malay: <https://youtu.be/8XjzhhWCI-o>
4. Tamil: <https://youtu.be/Wb94XfzI3oI>
5. Filipino: <https://youtu.be/4qqI5RRZfR4>
6. Thai: <https://youtu.be/qIC8h2uzuvo>
7. Japanese: <https://youtu.be/Ze3zHPEK8cw>
8. French: <https://youtu.be/qZoU0XqOLOU>
9. Spanish: <https://youtu.be/N7mg3csGOHc>
10. Portuguese: <https://youtu.be/SngiuKVRwac>

**Supplementary Video 2.** Demonstration of incorrect responses to a highly specific French question during multi-lingual assessment: <https://youtu.be/HJLxyXpBHQY>

**Supplementary Table 1**. Sources and location of medical content used to create the DR-COVID training dataset.

| **Source** | **Source location** |
| --- | --- |
| World Health Organization | https://www.who.int/emergencies/diseases/novel-coronavirus-2019 |
| Medscape | https://www.medscape.com/resource/coronavirus |
| National Health Service | https://www.nhs.uk/conditions/coronavirus-COVID-19/ |
| Ministry of Health, Singapore | https://www.moh.gov.sg/COVID-19 |
| Centre for Disease Control and Prevention | https://www.cdc.gov/coronavirus/2019-ncov/index.html |
| Mayo Clinic | https://www.mayoclinic.org/diseases-conditions/coronavirus/symptoms-causes/syc-20479963 |
| Gavi | https://www.gavi.org/vaccineswork/covax-explained |
| National Institute of Health | https://COVID19.nih.gov/ |
| Coalition for Epidemic Preparedness Innovations | https://cepi.net/ |
| European Respiratory Journal | https://erj.ersjournals.com/content/55/4/2000607 |

**Supplementary Table 2.** Global test questions used in assessment of user interface for DR-COVID and other publicly available question-answer chatbots.

| **Global test questions** | |
| --- | --- |
| How can I get a test for COVID-19? | Can COVID-19 stay on surfaces for very long? |
| How infectious are discharged COVID-19 patients? | Can I get exposed to the COVID-19 virus from food? |
| What takes place when a person gets infected with COVID-19? | What happens if I need to go cut my hair? |
| What is the case number for today? | What if I cannot practise 1m separation due to the crowd? |
| Which mask gives the most protection against COVID-19? | Can I use hydroxychloroquine to cure COVID-19? |
| In the long-term, will COVID-19 affect us? | Which are the different kinds of COVID-19 strains? |
| Which symptoms should I look out for that show I may have COVID-19? | Can breastfeeding woman receive COVID-19 vaccine? |
| Is the COVID-19 PCR accurate? | What vaccine will I be getting? |
| How many people do we need to vaccinate in order to achieve herd immunity? | Does immunity develop after vaccination? |
| I have multiple chronic illnesses. Is the vaccine still safe for me to take? | What advice is currently given for travel? |

**Supplementary Table 3.** Singapore-centric, and global test questions used in performance assessment for DR-COVID question-answer retrieval.

| **Singapore-centric test questions** | |
| --- | --- |
| Are the PHPC subsidies already applied for eligible participants? | How are the wet market restrictions decided? |
| Do I need to fill in a form to get subsidies at the PHPC? | In what way did the government decide the wet markets to impose these limitations on? |
| Is there a hardcopy application form to apply for PHPC subsidies? | Why are some wet markets deemed to be hot spots? |
| How do I apply for my family to continue doing to centre-based rehabilitation? | How can I find out whether my work is an Essential or Non-Essential Service? |
| What are the rules on centre-based rehabilitation? | Is my business an essential one? |
| Can I apply for home therapy for my family members? | How do I confirm that I have an essential business? |
| Am I allowed to use the exercise facilities at the hotel whilst I am serving SHN? | is PHPC covered under government subsidies? |
| What are the rules on using the facilities at the hotel whilst I am serving SHN? | Who bears the cost of a PHPC visit? |
| Are there restrictions on using the exercise facilities at the hotel during SHN? | What is the cost of treatment at a PHPC? |
| Will I be provided with food during the SHN at the designated facility? | Can I receive multiple PHPC subsidies if it visit more than one PHPC clinic on the same day? |
| Who will provide food for me during SHN? | Can I be subsidized many times in a single day for my PHPC visits? |
| Are there housekeeping services at the SHN designated facility? | What are the rules on PHPC subsidies? |
| Do these new variants exist in Singapore? | Who will fetch me from the airport to the Stay-Home Notice (SHN) designated facility? |
| Do these new variants appear in Singapore? | What are the ways I can travel from the airport to my Stay-Home Notice (SHN) designated facility? |
| Have we observed new virus strains here? | Who makes the transport arrangements from the airport to the SHN facility? |
| Can workers visit a person serving Stay-Home Notice (SHN) to repair utilities? | How will my family know where I am serving my SHN? |
| Can workers come to fix my home if I am under Stay-Home Notice (SHN)? | Who will inform my family members of my whereabouts whilst I am serving SHN? |
| Is it safe for utility workers to visit a person on Stay-Home Notice (SHN)? | Who updates my family on my SHN location? |
| May I host visitors at my SHN facility? | Will spot checks be done for people on quarantine? |
| Is my family allowed to visit me whilst I am serving my SHN? | How will I be monitored for compliance when on quarantine? |
| What are the rules on hosting visitors when I am serving SHN? | Does the government make home visits to monitor people on quarantine? |
| Can children continue to use common areas and facilities? | How do I know if a particular clinic is covered under PHPC? |
| What are the rules on playgrounds and common condo facilities for children? | Which clinic is involved in the PHPC scheme? |
| Are children allowed to use the common play areas? | Which clinics are participating in the PHPC program? |
| Is blood donation allowed after being vaccinated against COVID-19? | Will being on QO mean my medical leave is forfeited? |
| What are the rules on donating blood after being vaccinated? | Will my QO duration be deducted from my medical leave? |
| Is blood donation safe if I have been vaccinated against COVID-19? | What are the implications on my medical leave if I have to serve quarantine? |
| Do PHPCs supply complimentary masks? | Is there someone able to help me if I face difficulties coping? |
| Can I collect free masks under the PHPC scheme? | Is there a hotline for stress-related issues? |
| Does the PHPC scheme cover masks too? | Where can I seek respite for mental health issues? |
| Can I make a trip back home first, prior to going to the SHN facility? | What are the rules on per access arrangements? |
| How will I be transported from the airport to the SHN facility? | Am I allowed to visit my children if I am divorced? |
| Am I allowed to take a detour before heading to the SHN facility? | How can I find out more about per access arrangements? |
| Can I get out of my quarantine place? | Are homemade masks allowed for children? |
| What are the instances I can go out when on quarantine? | Am I allowed to adjust the masks given by the government to fit my child? |
| When can I be granted permission to leave my place of quarantine? | What are the rules on altering masks provided by the government? |
| Can I stay in a place other than my official NRIC address? | Is visiting permitted? |
| Am I allowed to stay in many places? | What are the rules on social gatherings? |
| What are the rules on living in different homes? | What social and family activities are permitted? |
| Under what circumstances can I remove my mask to smoke? | I got a call from MOH saying that they are calling me for contact tracing reasons. How do I check if it is real? |
| Am I allowed to smoke outside? | How do I check if the contact tracer is real? |
| Can I still smoke outside? | How do I protect myself from MOH scam calls? |
| Can I deliver groceries to my aged neighbours? | Does quarantine entitle me to a payment from the government? |
| Can I bring my elderly family member to buy daily necessities? | How can I apply for quarantine allowance for my employee? |
| Am I allowed to bring my elderly parent to see a doctor? | Do I get paid to serve quarantine? |
| Am I allowed to exercise or do sports outdoors? | Is a worker serving Stay-Home Notice (SHN) allowed to live in the same dormitory as others? |
| Are there any restrictions on sporting activities? | Is it safe for a worker to serve Stay-Home Notice (SHN) with others in a dormitory? |
| Are there any restrictions on exercising? | How do I keep other workers safe if someone is serving Stay-Home Notice (SHN) in the same dormitory? |
| Can I use my spouse's MediSave to pay for the subsidized payment at a PHPC? | Should I seek medical attention at the same PHPC if my flu does not go away? |
| How can I make payment for the PHPC's subsidized rates? | Is it advised to go to another PHPC? |
| Is Medisave allowed to offset the PHPC bill? | Am I allowed to doctor hop to another PHPC? |
| Is MOH able to provide more information on the confirmed cases location? I think that I have been at the same place as the confirmed cases, what should I do? | I live near a Government Quarantine Facility. Do I have to be worried? |
| Can I find out more details about confirmed COVID-19 cases? | Are quarantined people unwell? |
| What should I do if I have been in the same place as an infected person? | Does quarantine mean the person is infected? |
| Can my flatmates stay with me when I am on SHN? | Can I physically participate in workplace training? |
| What are the rules on living with others whilst I am on SHN? | Am I allowed to take part in training at the workplace? |
| Must I live apart from my flatmates if I get a SHN? | Are training programmes for essential workers permitted? |
| Does the government have access to my personal data? | What happens if a traveller has fever, cough, runny nose? |
| What are the Personal Data Protection Act (PDPA) policies surrounding COVID-19? | Should I visit the emergency department if I am sick after travelling? |
| Can companies collect, use and disclose personal data of visitors to places to allow contact tracing and other response measures if there is a COVID-19 case? | Is it appropriate to see a GP if I feel sick after returning from overseas? |
| If I share toilets during Stay-Home Notice (SHN), what should I watch out for? | Can I carry on delivering food to the elderly and needy? |
| How can I share toilets safely with someone on Stay-Home Notice (SHN)? | Am I allowed to deliver meals to the elderly? |
| Is sharing towels with a family member on Stay-Home Notice (SHN) permitted? | What are the rules on bringing food to the needy? |
| What rules should I follow when serving my Stay-Home Notice (SHN)? | How can unemployed people who are serving their quarantine get monetary assistance? |
| How can I serve my Stay-Home Notice (SHN) safely? | Will the government help the jobless who need to serve quarantine? |
| What activities are not allowed during my Stay-Home Notice (SHN)? | Can I get money if I am unemployed but need to serve quarantine? |
| Can Singapore citizens who need to travel overseas take priority for the vaccination? | What is the procedure of applying for the LOASP? |
| Can I get the vaccine first if I am a citizen and have to travel overseas for work or studies? | What documents are required to apply for LOASP? |
| Can non-Singapore citizens be vaccinated free of charge? | Can I break quarantine to attend to emergency matters? |
| Is vaccination complimentary for PRs, persons on Long Term Visitor Pass, Employment Pass, S Pass, Work permit, and dependant pass holders? | What are the consequences of going out when still on quarantine order? |
| Can I stay in the same room as someone serving their Stay-Home Notice (SHN)? | Am I allowed to go out during quarantine to settle emergency matters? |
| Am I allowed to share beds with someone on Stay-Home Notice (SHN)? | Will somebody take care of my car if I need to serve my SHN on arrival? |
| Is it safe to stay in the same room as my family member on Stay-Home Notice (SHN)? | Who brings my vehicle back if I arrive in Singapore via land and need to serve quarantine? |
| Can I receive discounts for non-respiratory illnesses? | What are the travel arrangements from the land checkpoints to the SHN dedicated facilities? |
| Will I be subsidized for a non-respiratory sickness? | Is it true that Pfizer vaccine causes deaths in elderly? |
| Can I get cheaper rates for non-respiratory illnesses? | Will Singapore stop vaccinating the elderly, as I heard Pfizer vaccine causes deaths? |
| Do I need to bring my CHAS card to the PHPC? | Do Pfizer vaccinations really cause deaths amongst the elderly? |
| What identity cards do I need to bring to get subsidies at the PHPC? | Is Grab allowed? |
| Which documents must I show at the PHPC to enjoy subsidies? | What are the rules on hitching a ride? |
| How do I know if I need to be on SHN? | Can I carpool? |
| How can I check whether I have a SHN? | Can I stay in the SHN facility for free? |
| Where can I clarify if I need to serve Stay-Home Notice (SHN)? | Is the stay in the SHN facility borne by the government? |
| Do I need a doctor to certify I can go back to work/ school after serving my Leave of Absence (LOA)/ Stay Home Notice (SHN)? | Who finances my stay at the SHN facility? |
| Is testing for COVID-19 required at the end of my quarantine? | Am I allowed to accompany my older family members out? |
| Must I get a swab to prove I am safe to work, after serving my SHN? | What are the rules on accompanying my elderly parent out? |
| Can I prebook the SHN facility prior to arrival? | Can I take my elderly family member out to walk? |
| Must I make a reservation for the SHN facility? | How do we determine who should serve their SHN in designated facilities? |
| Am I allowed to reserve a room at the SHN facility? | How shall we decide which Singaporeans should complete their SHN at the dedicated facilities? |
| Are bikers exempted from wearing a mask? | Are some Singaporeans allowed to not serve SHN at the designated facilities? |
| Can I ride my motorbike without a mask? | Can I remove my mask when engaging in strenuous exercise? |
| Under what circumstances can motorcyclists remove their masks? | What is considered strenuous exercise for allowances to be made to remove the mask? |
| Is there someone who can bring me essential supplies whilst I am serving SHN? | What are the criteria to decide whether an exercise in strenuous? |
| Is my family allowed to meet me to pass me items during my SHN? | Is wearing a mask compulsory at work? |
| Am I allowed to receive grocery deliveries whilst on SHN? | When am I allowed to remove my mask at the workplace? |
| Has there been animal-to-human transmission of COVID-19 in Singapore? | During which instances can I remove my mask at work? |
| Has COVID-19 infections occurred between humans and animals in Singapore? | What if my MC is longer than my entitled sick leave? |
| Do pets spread COVID-19? | Can I be granted more sick leave to cover my MC? |
| Is home personal care for the elderly still allowed? | What happens if my MC and sick leave do not tally? |
| What are the rules on home personal care? | Are home medical and nursing services allowed to continue? |
| Am I eligible to apply for home personal care during COVID times? | What are the rules on home medical and nursing services? |
| Who is eligible to continue receiving medical and nursing services at home? | What should I do if I run out of masks? |
| Can I interact with my elderly parents/ relatives? | Will I go to jail for not complying with mask wearing? |
| What are the rules on visiting elderly parents? | What are the punishments for non-compliance with mask wearing? |
| How do I ensure my parents living alone are not lonely? | What should we do if shops are too crowded? |
| Should I see my GP if I have respiratory symptoms? | Are we exempt from safe distancing if the supermarket is too crowded? |
| Is the PHPC clinic preferred over non-PHPC clinics if I have respiratory symptoms? | What are the rules on safe distancing if shops are crowded? |
| Can I go to my GP for regular follow up if it is now under the PHPC scheme? | Will a doctor come to see me if I fall sick when serving SHN? |
| Are GPs under the PHPC scheme open for non-COVID related visits? | Who will assist me in seeking medical attention when I am serving SHN? |
| Can my partner drop me off for groceries on the way to work? | How do I get medical aid during SHN? |
| What are the rules on dropping your spouse off to get groceries on the way to work? | How can I visit a doctor when serving my Stay-Home Notice (SHN)? |
| Are there places my partner can fetch me to, on their way to work? | In what way can I see a doctor when doing my Stay-Home Notice (SHN)? |
| Can you tell me the border restrictions for Singapore now? | How can I seek medical help whilst on Stay-Home Notice (SHN)? |
| Are short term visitors allowed to enter Singapore? | How will people under Stay-Home Notice get medical help? |
| Can people visit Singapore for a short while? | How can I look for help if my employer insists that I work a non-essential job? |
| What time do border restrictions take effect? | Who can help me if my employer forces me to work a non-essential job? |
| I am arriving in Singapore after the effective date and time. Can I still serve my SHN at home? | Are employers allowed to pressure me to do non-essential work? |
| How can I tell if the safe distancing ambassadors are real? | What will the Government do to enforce the measures? What are the penalties and who will be the ones to serve these enforcement penalties? |
| How do I tell if the enforcement officer is really appointed by the Government? Do I have to pay penalties upfront? | Will enforcement officers be able to give fines? |
| How can I check that safe distancing ambassadors are real? | Will I be punished for not adhering to safe distancing? |
| Do I need to pay penalties on the day itself? | Who is enforcing the rules on the ground? |
| When am I allowed to go outside? | Will families be assigned the same rooms at the SHN hotel? |
| When can I leave my home? | Are friends allowed to stay together at the SHN hotel? |
| For what purpose may I leave my house for? | Can my friends and I stay in the same room at the SHN hotel? |
| Are essential workers allowed to move around different areas in Singapore? | What are the rules for funerals now? |
| What can essential workers do to travel safely around Singapore? | What is the number of people who can go for a funeral? |
| How can I move safely around Singapore as an essential worker? | Are there any restrictions on funerals and wakes? |
| Can you tell me the precautions being taken at the borders? | Will people serving SHN be admitted to the hospital if they are found to be infected? |
| Who will be swabbed for COVID-19 at the border? | Can I call the ambulance if I have a medical emergency during my SHN? |
| What if a traveller has respiratory symptoms? | If I am diagnosed to have COVID-19 during SHN, what will happen to me? |
| How can you tell which traveller should get the COVID-19 swab? | Are there home delivery services for masks? |
| How will people who break the safe distancing rules be disciplined? | Do the SHN facilities and GQF function similarly? |
| What do you do if the traveller doesn't want to go for testing? | How is QO, LOA and SHN different? |
| What are the consequences if a traveller refuses to be tested? | When will Stay-Home notices be issued to travellers and how will it be done? |
| Will travellers get prosecuted if they don't want to get tested? | Who will need to serve the stay home notice? |
| Can you tell me what is a Community Isolation Facility? In what ways is it different from a Government Quarantine Facility? | Is the Stay-Home Notice a paper that is given to me? |
| What is the definition of a Community Isolation Facility (CIF)? | What time do CCs open? |
| What is the purpose of a Community Isolation Facility (CIF)? | When can I visit a CC? |
| What is the use of PHPC? | When can I go to a CC to collect masks? |
| How does the PHPC work? | How is the 14-day isolation duration officially counted? |
| What is the function of PHPC? | How does the 14 days of isolation work? |
| Can you tell me the recommendations for travelling now? | How do I count the 14 days of isolation? |
| What is the government's stance on travelling overseas? | Do essential workers have to eat by themselves? |
| Where can I travel to? | In what places can essential workers eat? |
| Is the SHN facility and GQF similar? | Is it safe for essential workers to eat outside? |
| Do the SHN facilities and GQF serve the same purpose? | Who can tell me more about PHPC? |
| Can you explain the terms QO, LOA and SHN? | Is there a hotline for PHPC-related enquiries? |
| How do local, community and imported cases differ? | Where can I find out more about PHPC? |
| What do local, community and imported cases mean? | Who can advise me on the SHN if I have questions? |
| What is the meaning of local, community and imported cases? | Who can tell me more about SHN? |
| Should I interact with my family members if I am on quarantine at home? | Is there a hotline for SHN-related enquiries? |
| What actions should I take while on quarantine at home? | Why is PHPC being introduced now? |
| What rules should I follow when serving my Leave of Absence (LOA)? | How do I count the 14 days of isolation? |
| How can I serve my Leave of Absence (LOA) safely? | What is the purpose of activating the PHPC now? |
| What activities are not allowed during my Leave of Absence (LOA)? | How will the PHPC help address the pandemic? |
| What can I do if I feel very stressed from caring for an old person during this COVID-19 period? | Which symptoms are eligible for the PHPC subsidy? |
| Who can help if I am feeling very stressed from caring for someone? | Which medical issues are eligible for the PHPC subsidy? |
| How can I seek respite from caregiver stress? | What are the criteria to receive subsidies at the PHPC? |
| Which kind of financial aid can I get from the Vaccine Injury Financial Assistance Programme (VIFAP)? | Can Singapore citizens and Permanent Residents who live overseas get vaccinated? |
| How can the Vaccine Injury Financial Assistance Programme (VIFAP) help me with money? | Do Singaporeans and Permanent Residents who live overseas have a chance to be vaccinated? |
| How will the VIFAP help me? | Can I get vaccinated if I am a Singaporean but living overseas? |
| How is the government helping the elderly and vulnerable? | Can I be excused from staying at SHN if I have a medical condition that makes it difficult for me to do so? |
| What policies does the government have to help the old and needy? | How can workers provide service to many different places? |
| What is the government doing to help the old and needy? | Am I permitted to be deployed to different branches to work? |
| Are the hotels contaminated after being used for SHN? | Are essential workers allowed to serve various places or individuals? |
| How are hotels cleaned after being utilised for SHN? | Can you tell me the rules for working at different sites? |
| Are hotels used for SHN dirty? | What is the duration the government is providing facilities for SHN for? |
| Where can I apply for exemptions from SHN if I have medical conditions? | Will the government continue providing dedicated facilities for Stay-Home Notice (SHN)? |
| Am I allowed to serve my SHN at home if I have difficulties staying in the dedicated facilities? | When will the designated Stay-Home Notice (SHN) facilities be closed down? |
| **Global test questions** | |
| Do young children have a lower risk to COVID-19 than adults? | What can I do if I am too sick to breastfeed my baby? |
| Are children less likely to be infected than adults? | Are there alternatives if I am too sick to breastfeed my baby? |
| Is there a low risk for children to get COVID-19 compared to adults? | Can asthmatics be exempted from mask-wearing? |
| Do COVID-19 patients still spread the virus after they are discharged? | Is it mandatory for patients with lung problems to wear a mask? |
| Is it possible for discharged COVID-19 patients to still be infectious? | Must I wear a mask if I have certain medical conditions? |
| How infectious are discharged COVID-19 patients? | By taking the vaccine, will we be protected from the different strains of COVID-19? |
| Does mRNA vaccine modify the genes? | Will the vaccine fully protect us against all current and future strains? |
| Are mRNA vaccines a type of genetic modification? | Will the vaccine provide immunity against all strains of the virus? |
| Do mRNA vaccines change human DNA? | Will taking the vaccine protect us from the different strains of the COVID-19 virus? |
| Do the mRNA vaccines modify the genes in our DNA? | What test should I take to see if I had COVID-19 previously? |
| Are COVID-19 vaccines halal certified? | Is there a test that can diagnose previous COVID-19 infection? |
| Are the COVID-19 vaccines considered suitable for Muslims? | How can one diagnose previous COVID-19 infection? |
| Can Muslims take the COVID-19 vaccine? | Should I see a doctor if I have recent travel history and symptoms of flu? |
| Do vaccines target the new COVID-19 strains? | How do I go and see a doctor if I just came back from overseas and I am experiencing symptoms of COVID-19? |
| Will the vaccines be able to fight off against the new virus strains? | Is there a specific way to see a doctor if I just came back from overseas and I am experiencing symptoms? |
| Will the vaccines that we are taking now be effective against new virus strains? | If a woman got the first dose then later became pregnant, can she still get the second dose? |
| Will the vaccines be just as good against the new virus strains? | What happens if pregnancy occurs after the first dose of vaccine? |
| Do vaccines work against the upcoming viral strains? | What happens if pregnancy between the first and second dose of vaccine? |
| Any side effects from vaccination? | Will my travel insurance still cover me if I had my trip cancelled due to COVID-19? |
| What are the vaccine side effects? | Can I still claim insurance if I cancel my flight due to the COVID-19 pandemic? |
| Which side effects of the vaccine are most common? | Can I claim insurance if I had my trip cancelled due to the pandemic? |
| Will vaccinated people be less likely to transmit disease? | What is the efficacy of the vaccine after a single dose? |
| Are vaccinated people less infective? | What is the effectiveness after one dose of vaccine? |
| Can vaccination curb the transmission of virus? | Is one dose of vaccine sufficient at protecting from infection? |
| What safe distancing measures should essential workers abide by? | Can I return to a non-essential business for maintenance purposes? |
| How can essential workers distance ourselves safely? | What are the rules on going back to a non-essential business for maintainance? |
| I am an essential service provider, what kind of safe distancing measures do I have to implement? | Am I allowed to return to my non-essential workplace to run errands? |
| Will adolescents be able to get sick with COVID-19? | Is taking the COVID-19 vaccine safe for people with chronic illnesses such as hypertension, high cholesterol and diabetes? |
| Can young people get infected with COVID-19? | Will I be able to take the COVID-19 vaccination if I have high cholesterol? |
| Will symptomless adolescents still be able to spread COVID-19 to others? | I have multiple chronic illnesses. Is the vaccine still safe for me to take? |
| Can asymptomatic young people spread COVID-19 to others? | Can the disease kill? |
| Will taking antiretrovirals be useful in preventing COVID-19 infection? | How high is the mortality rate for COVID-19? |
| Is the use of antiretrovirals possible in preventing COVID-19 infection? | Is it easy for COVID-19 to kill people? |
| Are antiretrovirals an effective treatment for COVID-19? | Will the disease kill a lot of people? |
| Is breastfeeding a possible route of transmission for COVID-19? | Is it possible for my friend or family help me to move house? |
| Does COVID-19 spread from the mother to baby through breastmilk? | Can I get someone to help me to move house? |
| Can females with COVID-19 still breastfeed? | What are the rules on asking friends and family to help me move house? |
| Can infected ladies continue breastfeeding? | Does my baby need to wear a mask? |
| Can COVID-19 transmission happen at our working place? | Can my infant not wear a mask? |
| Does workplace transmission of COVID-19 occur? | Is it okay for my infant to be exempted from wearing a mask? |
| Will COVID-19 infect me where I work? | What can I do if my child cannot tolerate wearing a mask? |
| Will other organs get damaged if we get COVID-19? | Can my child remove his mask if he feels hot? |
| Can COVID-19 hurt other organ systems? | Can my young child be excused from using a face mask? |
| How does COVID-19 harm organs other than the lungs? | Should I avoid places that have been visited by people with a COVID-19 infection? |
| Can I ask a repairman to fix my faulty electronics or utilities? | Should I be avoiding places where COVID-19 positive people have visited before? |
| Will I be able to call someone to come over to fix my household problems? | Is it true that we should avoid places where COVID-19 suspects have been to? |
| Are utility repairmen allowed to enter my home? | Can I live elsewhere if my place is too small? |
| Can people continue to come to my home to provide services? | What are the alternatives if my home is too small for everyone to live in? |
| Can I still get a tuition teacher to come over to teach my child? | What are the rules on staying elsewhere if my home is too small? |
| Am I able to donate blood after receiving the COVID-19 vaccine? | What if my family member's condition will worsen if he does not go for centre-based rehabilitation/ home therapy? |
| What are the rules on blood donation after getting vaccinated? | What are the rules on continuing centre-based rehabilitation/ home therapy? |
| Am I allowed to give blood after receiving the COVID-19 vaccine? | I need to continue attending centre-based rehabilitation in person, what do I do? |
| Can I get exposed to the COVID-19 virus from food? | Are we permitted to visit our relatives' homes to assist them? |
| Can food or drink items spread COVID-19? | Can we visit houses of our extended family to do caregiving? |
| Do certain food items spread COVID-19? | Can my parents visit and take care of their grand children? |
| Is it possible for me to get infected with COVID-19 by taking drinking water? | Can my relatives come over to help to take care of my child? |
| How likely is it for COVID-19 to spread through drinking water? | Can I visit my elderly parent regularly so they do not become bored? |
| Is it possible to get infected with COVID-19 by swimming in a public pool? | What sort of mask should be used to prevent COVID-19 spread? |
| I swam in the sea recently, is it possible fo me to fall sick with COVID-19? | Which mask gives the most protection against COVID-19? |
| Is it possible to get infected with COVID-19 by coming into contact with sewage with it? | What type of mask is the best to use for protection against COVID-19? |
| Will I fall sick with COVID-19 if I came into contact with wastewater? | In which situations am I permitted to go outside? |
| Does sewage or fecal matter spread COVID-19? | When am I allowed to go outside my home? |
| Is going for surgery after taking the vaccination still possible? | What kind of circumstances will allow me to leave the house? |
| Is it safe to undergo surgery after being vaccinated? | How do we know that the vaccines for COVID-19 have been well studied, considering how fast it was developed? |
| Are procedures allowed after vaccination? | How can we ensure the science is right when the vaccines were made so quickly? |
| Can I go outside to pick up my household member from shops outside? | Did they sidestep any processes when making the vaccine? |
| Can I go out to pick up a family member living with me from the supermarket? | What is the government's advice for essential workers who need to travel around the country? |
| Am I allowed to pick up my flatmate on the way? | Can you give me advice on the movements of essential workers? |
| Can I go outside to exercise with more than one person living together with me? | Do you know what are coronaviruses? |
| Can I go out to exercise with two other friends? | What is the meaning of COVID-19? |
| How many people can exercise outside together? | Can you tell me more about COVID-19 and the family of coronaviruses? |
| Is it possible for me to move house? | Can you tell me about the different types of vaccines? |
| Will I be able to move to my friend's house? | Can you tell me the different types of vaccines out there? |
| What are the rules on moving house? | What are the forms of vaccine? |
| As an essential worker, is it advised for me to move house to protect my family? | What is the number of vaccines we can take? |
| I want to reduce my risk to my family by moving out as I am an essential worker. Can I do so? | Can you tell me the different types of virus strains? |
| Can I get a non-COVID-19 vaccine together with a COVID-19 vaccine? | How do we tell the different virus strains apart? |
| Can I be simultaneously vaccinated with the COVID-19 and another vaccine? | Tell me about the different viral strains |
| Can I take two vaccines together, one of which is the COVID-19 vaccine? | What are the similarities and differences between the viral strains? |
| Can I take off my mask to wipe my face? | Which are the different kinds of COVID-19 strains? |
| Am I allowed to remove my mask outside to eat? | In what way are the COVID-19 strains different? |
| Are mealtimes an exemption from mask-wearing? | Can you give me the names of the different types of virus strains? |
| When should I put back my mask after eating outside? | Can you tell me the differences in the different types of virus strains? |
| Is it possible for me to send my employees to the doctor to get tested after their 14 days of isolation just to be sure? | Can you tell me all the different types of virus strains that are out there now? |
| I would like to send my students to check if they have COVID-19. Would that be possible? | How different are the variants of the COVID-19 virus? |
| Is a swab needed for students before returning to school at the end of 14 days? | What is inside the Pfizer vaccine? |
| Can i follow my elderly parent who is not living with me to see the doctor or get daily necessities? | Can you give me a list of all the ingredients inside the Pfizer vaccine? |
| Can I follow my elderly parent who lives in a different house for their errands? | What is inside the Pfizer vaccine? |
| Can I see my elderly neighbour to ensure he is doing well? | Can you name some risk factors of COVID-19? |
| Is it okay for me to check in on my neighbour who is living alone? | What activities predispose someone to get COVID-19? |
| How can I ensure my elderly neighbour is coping well? | What are the factors that increase the risk of catching COVID-19? |
| Am I allowed to go outside to exercise and do recreational activities? | In the long-term, will COVID-19 affect us? |
| Is it alright for me to go out to exercise? | What are the long term complications of COVID-19? |
| Is outdoor sports permitted? | Can you tell me some severe complications that can arise from COVID-19? |
| Will I be able to use proof of vaccination to travel? | What are the serious adverse effects of COVID-19? |
| Does proof of vaccination work for travelling? | Can you tell me the symptoms of COVID-19? |
| Am I allowed to travel with proof of vaccination? | What are the indications that show that I have COVID-19? |
| Am I allowed to hire a nanny? | Which symptoms should I look out for that show I may have COVID-19? |
| Can a nanny continue to visit to take care of my kid? | What sort of areas should we prioritise for disinfection in a non-healthcare setting? |
| Can I have a nanny to come over to take care of my child? | What are the priority areas that we should disinfect in a non-health care setting for COVID-19? |
| Does consuming probiotics help in the prevention of COVID-19? | Which areas should we disinfect first in a non-healthcare setting? |
| Will I be protected from COVID-19 if I keep consuming probiotics? | Can you explain to me immunity in the context of COVID-19? |
| Are probiotics proven to prevent COVID-19? | What is the science behind immunity from COVID-19? |
| Can people who need to travel for work or studies be prioritised to receive vaccination? | Can you tell me the things we know regarding immunity from COVID-19? |
| Can people who need to travel for work or studies become vaccinated earlier? | Through what method can the COVID-19 virus bind to cells? |
| Are Singaporeans who have to travel eligible for early vaccination? | What method does the COVID-19 virus use to attack and bind to cells? |
| Can I take care of someone who is COVID-19 positive at home? | What method does the COVID-19 virus carry out to bind to our cells? |
| Can I take care of someone who received a positive COVID-19 test at home? | What takes place when a person gets infected with COVID-19? |
| Can COVID positive patients receive care at home? | What happens in the respiratory tract when COVID-19 infects it? |
| After getting vaccinated is it possible to get COVID-19? | What goes on when a COVID-19 infection occurs? |
| Will people who received the vaccine still get infected? | Through what mechanism does the COVID-19 virus infect the respiratory tract? |
| Does immunity develop after vaccination? | What occurs when the virus infects someone? |
| I recently got two dose of the vaccine and am now fully vaccinated. Can I still get infected? | What is the pathogenesis of COVID-19? |
| Can COVID-19 lead to a higher incidence of blood clots? | Through that mechanism does the COVID-19 virus infect the respiratory system |
| Is it true that COVID-19 causes blood clots? | What goes on when COVID-19 infects someone? |
| Can COVID-19 increase risk of clotting? | What method does the COVID-19 virus use to attack the respiratory tract? |
| How likely is it to get chronic fatigue after recovering from COVID-19? | What occurs when COVID-19 virus invades the body? |
| Will I become chronically tired due to COVID-19? | What occurs when the virus attacks our respiratory tract? |
| Is it true that one can experience chronic fatigue after COVID-19? | What allows the COVID-19 virus to bind to cells? |
| Will the use of dexamethasone be able to treat COVID-19? | How can the virus adhere to cells? |
| Can I use steroids to cure COVID-19? | What happens if the supermarket/ convenience/ F&B outlet shop has too many visitors for me to follow safe distancing? |
| Is the use of steroids to treat COVID-19 supported by evidence? | What if I cannot practise 1m separation due to the crowd? |
| Will the use of hydroxychloroquine be able to treat COVID-19? | What should I do if safe distancing is impossible at a crowded market? |
| Can I use hydroxychloroquine to cure COVID-19? | What happens if I need to go cut my hair? |
| Is the use of hydroxychloroquine to treat COVID-19 supported by evidence? | What do I do if I need to get a haircut? |
| Will the use of immunomodulators be able to treat COVID-19? | What are the rules on getting a haircut? |
| Can I use immunomodulators to cure COVID-19? | What happens if I need to get new spectacles? |
| Is the use of immunomodulators to treat COVID-19 supported by evidence? | What do I do if I need my spectacles replaced? |
| Will the use of lopinavir/ritonavir/protease inhibitors be able to treat COVID-19? | Can I go to an optician shop? |
| Is the use of protease inhibitors to treat COVID-19 supported by evidence? | How many people do we need to vaccinate in order to achieve herd immunity? |
| Can I use protease inhibitors to cure COVID-19? | What percentage of the population has to get the vaccine in order for us to reach herd immunity? |
| Will the use of remdesivir be able to treat COVID-19? | The media keeps talking about herd immunity. Can you tell me what is it? |
| Can I use remdesivir to cure COVID-19? | Can you define "herd immunity" for me? |
| Is the use of remdesivir to treat COVID-19 supported by evidence? | Can you tell me more about COVAX? |
| How can I apply for home elderly care to continue? | What does COVAX stand for? |
| How can I continue to apply for the home personal care service for seniors? | Please explain COVAX to me |
| How can home care services for the elderly continue? | Can you tell me more about CEPI? |
| How certain are we in the COVID-19 PCR accuracy? | Can you tell me what is CEPI? |
| Is the COVID-19 polymerase chain reaction test reliable in its accuracy? | What is CEPI used for? |
| Is the COVID-19 PCR accurate? | What advice is currently given for travel? |
| Accuracy of COVID-19 PCR? | Can you give me the latest public health travel advisory? |
| Can you give me an overview of the differences between COVID-19 and influenza viruses? | Are there recommendations against travelling? |
| How different is the flu virus compared to COVID-19? | How different are pre-symptomatic people compared to asymptomatic? |
| Can you give me an overview of the similarities between COVID-19 and influenza viruses? | Do pre-symptomatic and asymptomatic people have np symptoms? |
| How similar is the flu virus compared to COVID-19? | How do I know what is the difference between pre-symptomatic and asymptomatic people? |
| How does proper ventilation control the spread of COVID-19? | Is there a guideline to follow for disinfection of outdoor spaces? |
| What is the role of ventilation in preventing the spread of COVID-19? | Could you guide me on how to conduct disinfection of outdoor spaces? |
| What is the usefulness of ventilation in COVID-19? | What is the correct protocol for disinfection of outdoor spaces? |
| How do other types of virus strains come about? | What role do children play in the transmission of COVID-19? |
| How do various viral strains arise? | How do children play a part in the transmission of COVID-19? |
| How do the viruses produce new and different strains? | Do children transmit COVID-19 more than adults? |
| What leads to the different virus strains to come into existence? | Can COVID-19 be treated by antibiotics? |
| What should I do to get tested for COVID-19? | Do antibiotics work to cure COVID-19? |
| How do I go about testing for COVID-19? | What are the treatment methods available for COVID-19? |
| How can I get a test for COVID-19? | How should the treatment of COVID-19 go? |
| How do vaccines like the Pfizer mRNA vaccine work? | What can I do if I get side effects from vaccination? |
| What happens when using a mRNA vaccine? | Should I seek medical attention if I suffer from side effects of the vaccine? |
| What is the mode of action of mRNA vaccines? | How long should observation of side effects be? |
| What does an mRNA vaccine contain? | What are the activities that vaccinated people can do? |
| What is the process of COVID-19 rapid tests? | Are vaccinated people allowed to do more activities? |
| What is the mechanism of action of rapid COVID-19 tests? | Can vaccinated people do more activities than unvaccinated people? |
| What is the science behind rapid COVID-19 tests? | Can you tell me the type of test that I should take to see if I have COVID-19? |
| How long can COVID-19 remain on surfaces? | What test is appropriate to diagnose COVID-19? |
| What is the length of time the virus can live on surfaces? | Which test can diagnose COVID-19? |
| How does the spreading of COVID-19 take place? | Which types of mask should we wear? |
| Can I get COVID-19 by shaking someone's hand? | Do I have a wear an N95 mask? |
| What is the method of spread for COVID-19? | Can I wear a cloth mask? |
| How long can COVID-19 live on surfaces? | What varieties of masks are recommended? |
| Can COVID-19 stay on surfaces for very long? | What kind of vaccine is recommended? |
| Does the COVID-19 virus remain on surfaces for very long? | Which vaccine does the government recommend? |
| How do people get infected with COVID-19 through surfaces? | Can I pick the vaccine to take? |
| How does COVID-19 infect through surfaces? | Can you tell me the vaccines that are available now? |
| What is the transmission method for COVID-19 through surfaces? | Currently, what are the vaccines that are available? |
| What is the route of transmission for COVID-19 to spread through surfaces? | Can you tell me about the vaccines available now? |
| In what way does weather affect the transmission of COVID-19? | What vaccines are in stock? |
| Can you tell me how does the weather affect COVID-19 transmission? | Which vaccines are accessible now? |
| How does hot weather affect COVID-19 transmission? | How do I know whether I should take a test for COVID-19? |
| In what way does the climate affect the infectivity of COVID-19? | When is testing for COVID-19 useful? |
| How powerful is the vaccine? | When is appropriate to get tested for the virus? |
| How good is the vaccine? | Where can people eat? |
| What is the effectiveness of the vaccine? | Which places are appropriate for essential workers to eat? |
| How fast can rapid antigen tests work? | Where would be alright for people to have their meals? |
| How soon can I get a result from the rapid antigen test? | Where can I go to to find out the latest information on the COVID-19 situation? |
| Speed of rapid antigen tests? | Can you direct me to the place where I can get the latest information on the COVID-19 situation? |
| How will the Pfizer vaccine be administered to people? | Can you tell me where I can go to find out the latest information on the COVID-19 situation? |
| Where will the Pfizer vaccine be injected into? | What group of people with a risk of anaphylaxis should not be vaccinated? |
| Through which route is the Pfizer vaccine given? | Who is at risk of anaphylaxis and should not take the vaccine? |
| How much time do I have to wait before taking other vaccines? | Who is vulnerable to anaphylaxis and should not receive the vaccine? |
| After taking the COVID-19 vaccine, what is the waiting duration before I can take other types of vaccine? | Can you tell me the types of people that have an anaphylaxis risk and should not take the vaccine? |
| What is the safe duration between the COVID-19 vaccine and other vaccines? | Who are the prioritised groups, and why do we prioritise them? |
| What is the estimated time to cook food to remove the COVID-19 virus? | Why are some people given the vaccine earlier than others? |
| What is the minimum duration needed to cook food to ensure it does not have COVID-19? | For which populations does vaccination take precedence? |
| Can cooking food break down COVID-19? | What side effect will take the longest to show? |
| Can you tell me how long it takes for the virus to incubate? | Which vaccine side effect is the most chronic? |
| What is the incubation period for the COVID-19 virus? | What is a late side effect of the COVID-19 vaccine? |
| What is the meaning of incubation period? | Can you tell me what sort of surface disinfectants will be effective in combating COVID-19? |
| What is the isolation period people should take if they have COVID-19? | Can you give me a list of surface disinfectants that will be good to use against COVID-19 in non-health care settings? |
| If we have COVID-19, how long should we remain at home and be isolated? | Which is the best disinfectant to clean surfaces? |
| How long should an infected person stay at home before it is safe to go out? | Can I choose to take the Pfizer vaccine? |
| What are the active cases now? | Am I allowed to take only the Pfizer vaccine? |
| How many people are currently having an active infection? | How do I know which vaccine to choose? |
| What is the case load now? | Which vaccine will be administered to me? |
| How many cases of COVID-19 are there in the world? | Am I allowed to select the brand of vaccine? |
| Worldwide case numbers today? | What vaccine will I be getting? |
| Worldwide what is the case number now? | Can I exercise preference for which COVID-19 vaccine to receive? |
| What are the number of active COVID-19 infections? | Has the Sinovac vaccine gained approval? |
| How many cases are active now? | Which vaccines have gained approval for use? |
| What is the case number for today? | What is a list of all the approved vaccines? |
| How many people are currently having an infection today? | What are the special circumstances in which we can go outside? |
| What is the current worldwide case count? | Are there any exemptions to leaving the house with other people? |
| How many people are currently having an infection worldwide? | Under which circumstances can we leave the house with somebody else? |
| What are the number of active COVID-19 infections today? | Can breastfeeding woman receive COVID-19 vaccine? |
| Case number today? | Is breastfeeding a contraindication to vaccination? |
| Today got how many cases? | I recently gave birth to a child and I am now breastfeeding. Can I still get the vaccine? |
| Can you tell me at the moment, how many people are vaccinated? | Who cannot get the COVID-19 vaccine? |
| How many people have been vaccinated so far? | Is the vaccine for everyone? |
| How many people have received the vaccine thus far? | Which groups of people are unsafe for vaccination? |
| What is the number of people who received the vaccine? | Who absolutely cannot get vaccinated? |
| During this time of COVID-19, how should I be washing my fruits and vegetables? | Can we still get vaccination even after being infected with COVID-19? |
| How long should I wash fruits and vegetables to reduce the risk of COVID-19? | Can previously infected people take the vaccine? |
| Is there a specific method of washing fruits and vegetables during this COVID-19 period? | Can a pregnant woman receive the vaccine? |
| Is there a guide to wearing a mask? | Why is wearing a mask important? |
| Is there a specific way to wear a mask? | Do I have to wear a mask? |
| What is the correct way to wear a mask? | Is it mandatory to wear a mask? |
| How long after vaccination can a woman try to conceive? | Is wearing a mask necessary? |
| How long after vaccination can a lady become pregnant? | Why are the different types of strains important to us? |
| What is the duration a lady needs to wait before getting pregnant? | Why should we be so concerned about there being different types of strains? |
| I just finished getting my second dose of the Pfizer vaccine. When can I start to try conceiving? | What is so important about knowing about the different types of virus strains? |
| What will the government do to help people who experiences serious side effects after vaccination? | Why do we need to know about the different virus strains? |
| Will the government compensate people who get severe reactions from the vaccine? | How are different viral strains significant? |
| Are there policies that help people who suffer serious vaccine side effects? | Will public health measures such as rostered routine testing (RRT), acute respiratory infection (ARI) and personal protective equipment (PPE) still be required by staff who are vaccinated? |
| I was unable to breastfeed due to falling sick from COVID-19. When is a good time to start breastfeeding again? | Are vaccinated people exempt from wearing PPE? |
| When can I restart breastfeeding after recovering from COVID-19? | Is it true that vaccinated people do not have to do regular ART? |
| How long after my COVID-19 infection should I start breastfeeding? | Can we choose the brand of vaccine we get? |
| Can I continue regular gatherings with my friends and relatives? | Can we choose the type of vaccines to take? |
| Can I do a get-together with a group of friends? | Do we have a choice of vacicne to receive? |
| Are parties allowed? | Will public health measures such as personal protective equipment (PPE) for healthcare workers still be needed after deploying COVID-19 vaccines? |
| Can I go outside if I am given a medical certificate (MC) by the PHPC? | As vaccination rates are high, do healthcare workers still need to wear protective equipment? |
| I just got medical leave from my doctor. Will I still be able to go out to buy food? | Is PPE necessary for healthcare workers if our population has high vaccination rates? |
| What are the rules on going out if I am on MC? | As there are new rules that do not permit working at different branches, can I carry on providing healthcare to different people or places? |
| I am feeling too sick to breastfeed my baby directly. What do I do? | Can I provide my healthcare services to multiple areas? |
| Will we be tested for antibodies to deem that vaccination was effective? | What are the rules on providing healthcare services to many branches? |
| Is post-vaccination testing for antibodies useful? | Will there be monitoring of staff and residents for antibodies post-vaccination? |

**Supplementary Table 4.** Additional questions of the English language from collaborators.

| How long does one take to recover from COVID? | Is COVID curable? |
| --- | --- |
| Where do I go if my ART test is positive for COVID? | What are the chances of dying from COVID? |
| Is the 3rd dose of vaccination effective? | Does hydroxychloroquine work for COVID? |
| When will COVID end? | Does COVID affect children? |
| Where to vaccinate? | How does vaccine work? |
| Can I get reinfected? | What are side effects of the vaccine? |
| Who can get COVID? | Is the third dose vaccine needed? |
| Why is COVID so difficult to eradicate? | Does COVID cause infertility? |
| How long does COVID stay in the body? | Why did COVID happen? |
| What are the chances of being reinfected with COVID? | Is there a symptomatic and asymptomatic phase for COVID? |
| How reliable is COVID ART? | What are the different strains? |
| Does COVID cause brain damage? | Who is most at risk for COVID? |
| Does COVID cause blindness? | How do I know I have COVID? |
| Is COVID getting more deadly? | How long to recover from COVID? |
| How many people did COVID kill? | Can I be asymptomatic? |
| When would COVID end? | Can I dine out? |
| Who needs to be on quarantine order? | Can I travel? |
| Which strain is the worst? | How did COVID start? |
| Can I get COVID twice? | What are the different strains of COVID? |
| Will COVID stop? | Why do I need to wear mask? |
| Why do I need to take the COVID vaccine? | What are the side effects of COVID? |
| Is mask 100% safe? | Which country has most COVID cases? |
| Where can I get mask from the government? | Are children at lower risk of COVID-19 than adults? |
| Can I go out in groups of 8? | What are the risk factors for COVID-19 |
| What is the quarantine timeline in Singapore? | How many cases of COVID are there |
| Is a booster shot required? | How easily will I get COVID |
| Can I exercise after getting COVID? | Is the vaccine useful? |
| Is it safe to exercise after a COVID vaccine? | Can I get a vaccine if pregnant? |
| When should I get a vaccine? | What is the difference between ART and PCR? |
| Where can I change my trace together? | Can I go for a wedding dinner? |
| Is COVID like SARS? | Is COVID dangerous? |
| Can COVID cause eye redness? | Can COVID be cured? |
| Does Ivermectin work for COVID? | Among people with COVID, how many actually die? |
| What is delta variant, is it that bad? | How to avoid COVID? |
| What are the risks of mRNA vaccines for pregnant women? | Where did COVID come from? |
| Can I take diclofenac for fever after the COVID vaccine? | If I miss my Pfizer vaccine dose by more than 2 months, can I still take the vaccine dose? |
| Does COVID cause stroke? | Why do some people have more severe COVID illness? |
| If I had COVID before is the vaccine useful? | Is COVID worse in children? |
| Can COVID infection affect lung function? | Can pregnant women pass COVID to the child? |
| Does COVID infection cause fits? | How long after someone is exposed to COVID do they fall sick? |
| Can I take two different COVID vaccines? | Is it possible to catch COVID if I am always at home or wearing a mask? |
| How do I know which strain I am infected with? | Is ART the same as PCR? |
| How often do we need to vaccinate? | Is the third dose of vaccine necessary? |
| Is vaccination compulsory? | Can I take the vaccine if I am preparing to get pregnant? |
| When can we stop wearing masks? | Is COVID same as common cold? |
| Can my friends QO together? | I am worried case numbers are rising? |
| Do I need a booster jab after full vaccination? | Are we having a lockdown again? |
| Who is eligible for Home Recovery? | Can the swab test tell which strain I have? |
| My ART is positive. What do i do? | How are asymptomatic patients diagnosed? |
| How many people can dine in together? | What mask is the best? |
| Can I take the COVID vaccine booster dose? | Can COVID spread through eyes? |
| Where can I get my COVID vaccine? | What is protection rate from vaccines? |
| What group size is safe? | Who will die from COVID? |
| Is the hospital safe to visit? | How do I know if I have COVID? |
| What does endemic mean? | What are some COVID symptoms? |
| What should I do if I am diagnosed with COVID? | What are the severe complications of COVID-19? |
| Do I need COVID vaccine booster? | What are coronaviruses? |
| Is mask wearing effective in preventing spread of COVID? | What is the treatment for COVID-19? |
| What can I do during SHN? | Can I donate blood after receiving the COVID-19 vaccine? |
| Is alfresco safer than indoor dining? | Is COVID-19 vaccination safe for those with chronic illness? |
| What should I do if I am tested positive? | Do I get subsidies for the treatment of other non-respiratory illnesses? |
| I was exposed to COVID-19 case, what should I do? | Home personal care will be scaled down to serve only seniors with inadequate family support. How do I apply for continuation of service for these seniors? |
| How often should I use the ART? | How much do I have to pay when I go to a participating PHPC, and what treatment can I expect to receive? |
| How much are the ART kits? | I heard that people who are served a Quarantine Order (QO) will receive $100 a day under the Quarantine Order Allowance (QOA). Is that true? |
| How accurate is the ART kit? | Do I need to be on Stay-Home Notice (SHN)? |
| If my ART result is negative, do I need to test again? | I have been given medical leave by the PHPC doctor. Can I still go out to run my errands? |
| Will my PCR/ ART test be positive after taking the COVID-19 vaccine? | How does COVID spread? |
| Do I need antibody testing after COVID-19 vaccination? | What is the delta variant? |
| How can 1 get COVID? | Are my dogs at risk of COVID? |
| What to do if I have COVID? | When should I do a COVID PCR test? |
| Does prone ventilation work for COVID? | What are complications of COVID? |
| Must children wear mask? | What masks are effective against COVID? |
| Where are the PHPCs? | What is the treatment for COVID? |
| Can I go to church? | Who is at high risk of vaccine allergy? |
| Where can I see a doctor? | Is it safe to get COVID vaccine during pregnancy? |
| What is a swab test? | Who is eligible for vaccination? |
| If I had COVID do I still need to be vaccinated? | Is herd immunity real? |
| Can COVID be treated at home? | Can COVID spread through kissing? |
| Does COVID cause permanent damage? | Do I need to wear a face shield? |
| I have history of asthma, is the COVID vaccine safe? | How do I know I have COVID? |
| What should I do if I test ART positive? | Can I take both Pfizer and Moderna? |
| If I am COVID positive, how will I be deemed to have recovered from COVID? | Can I take the Influenza vaccine with the COVID vaccine? |
| Which vaccine gives higher immunity against COVID? | Can I take the COVID vaccine? |
| Can I recover at home if I have COVID? | What are contraindications of COVID vaccine? |
| How to prevent spreading COVID? | Are children susceptible to COVID? |
| How serious is COVID-19? | Can COVID spread in restrooms? |
| How do I know that I have COVID-19 since tests can be false negative? | Can we start travelling? |
| Can my loved one be discharged from the hospital since my household members have high risk of exposure to COVID-19? | How often do I need to do antigen rapid test? |
| Can I take the COVID-19 vaccine? | Any antiviral effective against COVID? |
| Will I die from vaccine side effects? | What is the practice of social distancing? |
| Are there long term effects of COVID? | Can I stay with my friends? |
| What are the number of COVID cases today? | Can I organise a party at home? |
| Where can I get tested? | Can my children still go to tuition centre? |
| Is there treatment for COVID? | Will I die from COVID-19? |
| How to differentiate COVID from common flu? | When should I test for COVID-19? |
| When can I travel again? | Is the COVID-19 vaccine effective? |
| How many COVID variants are there? | What are the side effects of the COVID-19 vaccine? |
| Should i avoid going to places with previous COVID cases? | How good is the vaccine? |
| How long should I stay home after a positive ART test? | How to prevent COVID-19? |
| Can I die from COVID? | I am feeling tired, do I have COVID-19? |

**Supplementary Table 5.** Additional questions of 10 non-English languages from collaborators, and their corresponding questions in English.

| **Non-English language** | **Question in English** | **Question in non-English language** |
| --- | --- | --- |
| **Mandarin** | After recovering from COVID-19 infection, will I get infected again? | 感染冠病康复后，会被第二次感染吗？ |
|  | After vaccination, can I still get COVID-19? | 打了疫苗后还会感染新冠吗? |
|  | Are antibiotics effective in preventing or treating COVID-19? | 抗生素对预防或治疗COVID-19有效吗？ |
|  | Are badminton halls open now? | 现在可以去羽毛球馆吗？ |
|  | Are mRNA vaccines safe? | mRNA疫苗安全吗？ |
|  | Are the current vaccines effective against Delta strain? | 目前疫苗对delta病毒有效吗？ |
|  | Are there long-term effects of COVID-19? | COVID-19有长期影响吗？ |
|  | Are there measures or specific ways for workers who are quarantined at home to check their physical health? | 自行在家隔离的工人，是否有措施或具体途径检测身体健康状况？ |
|  | Are there new COVID-19 cases nearby? | 附近是否有新增案例？ |
|  | Are there treatments for COVID-19? | 有治疗COVID-19的方法吗？ |
|  | As a foreign worker, how to deal with the salary subsidy, meal and accommodation allowance during the isolation period? | 作为外籍工人, 如何处理隔离期间的薪水补助,餐食住宿津贴？ |
|  | As a foreign worker, I am under a lot of pressure and feel uncomfortable. Can I undergo counselling? | 作为外籍工人, 我的压力很大，心里不舒服。可以辅导吗？ |
|  | As a foreign worker, I feel unwell. What should I do? | 作为外籍工人, 我觉得身体不适。怎么办？ |
|  | As a foreign worker, under what circumstances can I resume work? | 作为外籍工人, 在什么情况下才能复工？ |
|  | Between men, women, and children, who is at greatest risk of infection? | 男人女人和儿童，谁最容易感染？ |
|  | Can children be infected? | 小孩会感染新冠吗? |
|  | Can I eat out now? | 我现在可以堂食吗？ |
|  | Can I go overseas for travel? | 我能否出国旅游？ |
|  | Can I go to a gym? | 我可以去健身房吗？ |
|  | Can I mix the type of vaccination? | 疫苗可以参类打吗？ |
|  | Can I receive a COVID-19 vaccine? | 我是否适合接种疫苗？ |
|  | Can infants be infected with COVID-19 too? | 幼儿也能感染到冠病吗？ |
|  | Can people get infected COVID-19 from animals? | 人会从动物那里感染COVID-19吗？ |
|  | Can pregnant ladies vaccinate? | 孕妇可以打疫苗吗？ |
|  | Can the elderly and young be vaccinated? | 老年人和小孩可以接种疫苗吗? |
|  | Did the third booster shot undergo clinical trials? | 第三支疫苗有经过学监吗？ |
|  | Do cats, dogs and other pets get infected and transmit COVID-19? | 猫狗等宠物会感染和传播病毒吗？ |
|  | Do vaccines prevent mutations in COVID-19? | 疫苗能防止冠状病毒变异株吗？ |
|  | Does COVID-19 booster cause cytokine storm? | 接种第三支疫苗之后感染新冠是否会出现免疫风暴? |
|  | Does COVID-19 infection affect lifespan? | 得了新冠治疗好了会影响人的寿命吗？ |
|  | Does COVID-19 significantly affect children 5 years and younger? | 新冠疫情对5岁以下的儿童影响大吗？ |
|  | Do second or multiple COVID-19 infections cause cytokine storm and higher mortality in the young? | 二次甚至多次感染是否会导致免疫风暴并导致在年轻群体中死亡率升高? |
|  | Does second or multiple COVID-19 infections occur? | 新冠是否会出现二次甚至多次感染? |
|  | Does wearing a mask prevent infection? | 戴口罩能避免被冠状病毒感染吗？ |
|  | During breastfeeding, can I take the vaccine? | 哺乳期可以打新冠疫苗吗？ |
|  | For cancer patients receiving chemotherapy, are they suitable candidates for the vaccine? | 癌症患者接受化疗，适合接受疫苗吗？ |
|  | From which countries do returnees to Singapore not have to be quarantined? | 从哪些国家来新加坡的旅客不需要隔离？ |
|  | How about eating out at restaurants? | 在餐厅吃饭怎么办？ |
|  | How can I eat outside safely? | 我怎么在外面吃安全？ |
|  | How can I prevent COVID-19 infection? | 如何预防新冠? |
|  | How can I register for vaccination booster shot? | 怎样登记接种疫苗加强针？ |
|  | How can I use the gym safely? | 我怎么在健身房锻炼安全？ |
|  | How can infected people seek emergency help? | 冠状病毒患者应何时寻求急救？ |
|  | How can the mutation of COVID-19 be effectively prevented? | 病毒变异如何有效阻断？ |
|  | How can we improve our immunity? | 怎么增强免疫力，有什么建议？ |
|  | How can we protect others and ourselves if we do not know who is infected? | 如果我们不知道谁被感染了，我们如何保护他人和我们自己？ |
|  | How can we reduce the rate of transmission? | 我们如何能减少感染到冠病的可能性？ |
|  | How do I know whether I am infected? | 如何得知自己是否感染新冠? |
|  | How does COVID-19 compare to the common cold? | 如何区别新冠与普通流感? |
|  | How does COVID-19 vaccine work? | 冠病疫苗如何发挥作用？ |
|  | How is COVID-19 transmitted? | 冠状病毒是如何传播的？ |
|  | How long after vaccination are antibodies produced? | 注射疫苗多久后会产生抗体？ |
|  | How long do I need to home isolate if I have COVID? | 得了冠状病毒需居家隔离多久？ |
|  | How long does it take to develop symptoms? | 出现症状需要多长时间？ |
|  | How long is the incubation period of COVID-19? | 新冠病毒的潜伏期有多久？ |
|  | How long will home recovery last? | 居家隔离什么时候可以结束？ |
|  | How many cases were there yesterday? | 昨天有多少新增病例？ |
|  | How many clusters are there in Singapore currently? | 新加坡现在有多少个感染群？ |
|  | How many community cases of COVID-19 infection were detected today? | 今天有多少社区确诊病例？ |
|  | How many deaths are there today? | 今天死了多少个人？ |
|  | How many people can gather as a group? | 一组最多能有几个人聚集？ |
|  | How many vaccines are there currently? | 目前市面上有多少种疫苗？ |
|  | How many vaccines can we take in Singapore now? | 新加坡目前可接种哪几种疫苗？ |
|  | How many variants of COVID-19 are there now? | 目前病毒有几种变异？ |
|  | How much does the PCR test cost? | 核酸检测的费用是多少？ |
|  | How much is the PCR test for COVID-19? | 做一次新冠核酸检测要多少钱？ |
|  | How often would pandemics like COVID-19 happen? | 这样的冠状病毒流行还会有多次爆发？ |
|  | How serious is COVID-19 infection? | 冠状病毒感染有多严重呢？ |
|  | How shall I know if I have been infected with COVID-19? | 我能怎么知道自己是否受到冠病感染？ |
|  | How to book the booster shot of COVID-19 vaccines? | 如何预约新冠疫苗加强针？ |
|  | How to deal with issues of legal exit of foreign workers whose work permits have expired due to the large number of flight cancellations without warning? | 航班无预警的大量取消，准证到期的外籍工人，合法出境问题要如何处理？ |
|  | How to identify COVID scam calls? | 怎么分别诈骗电话？ |
|  | How to prevent COVID-19 when living with people currently on sick leave, history of overseas travel and contact, home notification, or are quarantined? | 在与目前有病假/有海外旅行史和接触/有居家通知或被隔离的人生活时, 如何预防新冠病毒? |
|  | How to use these three Mobile applications: FWMOMCare, SGWorkPass and TraceTogether? | 该如何使用这三个手机应用程序: FWMOMCare 、SGWorkPass及TraceTogether（合力追踪）? |
|  | How to wear a mask? | 如何佩戴口罩? |
|  | I am a close contact of a positive case; do I need to do PCR test? | 我是一个确诊病例的密切接触者，我需要做核酸检测吗？ |
|  | I am a close contact of a positive case, what should I do? | 我是一个确诊病例的密切接触者，我应该怎么做？ |
|  | I am on home recovery; how can I consult a doctor? | 居家康复时怎么找医生？ |
|  | I am unable to be vaccinated due to allergies, can I still dine out? | 我对疫苗敏感，还可以外出用餐吗？ |
|  | I am waiting for transplantation. Will I get COVID-19 from the donor? | 我正在等待移植, 会从捐增者那感染新冠病毒吗？ |
|  | I feel giddy, what should I do? | 我头晕，怎么办？ |
|  | I have a fever, what should I do? | 我发烧了，该怎么办？ |
|  | I have a positive ART kit test result, what do I do next? | 冠病快速检测呈阳性，接下来应采取什么步骤？ |
|  | I have a stomach ache, what should I do? | 我肚子疼，怎么办？ |
|  | I have COVID-19 symptoms, what should I do? | 我有新冠的症状， 应该怎么做？ |
|  | I want to find out if I had COVID-19 in the past, what test could I take? | 我想知道自己是否得过COVID-19，我应该做什么检测？ |
|  | If I am a family of 3 with a young child, do I still dine in as groups of 2? | 家中有年有小孩，出外用餐还是得两个人吗？ |
|  | If I am infected, what should I do next? | 出现症状后我该怎么办? |
|  | If I am pregnant, can I take the vaccine? | 孕妇可以打新冠疫苗吗？ |
|  | If I am quarantined, what preventive measures should I take? | 若居家隔离，应采取什么预防措施？ |
|  | If I have COVID and elderly parents at home, are there government facilities for me to self isolate? | 得了冠状病毒但家里有年老的父母，政府有别的地方给我居家隔离吗？ |
|  | If I return to Singapore from abroad, do I need to be quarantined? | 我从国外回到新加坡需要隔离吗？ |
|  | If I wear a mask, am I still considered a close contact? | 如果戴口罩， 还属于密切接触者吗？ |
|  | If infected while pregnant, is the baby infected? | 孕妇感染的话对胎儿有影响吗？ |
|  | If infected with COVID-19, what are the treatment options? | 若被冠状病毒感染，有什么治疗方法？ |
|  | If infected, will pregnant ladies spread COVID-19 to their infants? | 孕妇患冠病，会传染给胎儿吗？ |
|  | If medical resources are overwhelmed and critically ill patients cannot be effectively treated, what is the mortality rate of COVID-19? | 在医疗资源崩溃情况下也就是重症患者无法得到有效救治，新冠病死率是多少? |
|  | If new workers cannot complete the application for white card within the validity period, can their stay be extended through the special pass? | 新工人入境白卡有效期内无法完成的issue手续，是否通过special pass延期？ |
|  | If the herd immunity is implemented, does it mean that most people will be infected multiple times? | 如果践行群体免疫政策，是否意味着大多数人会感染多次? |
|  | If landlords do not accept newly arrived workers, how are arrangements made for workers who need to isolate themselves? | 房东不接受新入境的工人，那么如何安置这些需要自行隔离的工人？ |
|  | In addition to fever, does COVID-19 have other common symptoms? | 除了发烧，新冠病毒有更其他常见症状吗？ |
|  | In many big cities, what are the reasons for multiple rounds of infection? | 诸多大城市，多轮感染的原因是什么? |
|  | In public spaces, is the infrared body temperature detector accurate? | 在公共空间, 红外线体温检测仪准确吗？ |
|  | Is COVID-19 the same as SARS? | COVID-19与SARS一样吗？ |
|  | Is dine-in allowed at hawker centres now? | 现在可以在食阁堂食嘛？ |
|  | Is fever still a powerful indicator of coronavirus disease? | 发烧是否还是预测冠病的有力指标？ |
|  | Is it necessary to sanitize my clothes upon returning home? | 回家以后需要给衣服消毒吗？ |
|  | Is it really better to be vaccinated? | 打疫苗真的有比较好吗？ |
|  | Is overseas travel allowed? | 我可以到国外旅行吗？ |
|  | Is the COVID-19 vaccine effective? | 冠病疫苗有效吗？ |
|  | Is the effect of COVID-19 on different people significant? | 新冠对于不同人种的影响大吗？ |
|  | Is there a vaccine for COVID-19? | 有针对COVID-19的疫苗吗？ |
|  | Is there any quarantine requirement when traveling back from the US? | 去美国旅游回来需要隔离嘛？ |
|  | Is there risk of transmission at public swimming pools? | 在公共泳池游泳会有感染新冠病毒的风险吗？ |
|  | Is traditional Chinese medicine useful against COVID-19? | 中草药对冠病有疗效吗？ |
|  | Is transmission possible during the incubation period? | 潜伏期间有传播的可能性吗？ |
|  | Is wearing a mask needed for outdoor activities? | 户外运动需要戴口罩吗？ |
|  | My ART kit test result is negative, but I have flu like symptoms; what precautions should I take? | 冠病快速检测呈阴性，但还是有流感症状，接下来应采取什么步骤？ |
|  | My ART result is positive, should I go to the hospital? | 我的自助抗原快速检测结果呈阳性， 我应该去医院吗？ |
|  | My entire family is on home recovery, how should we buy daily necessities? | 我们全家都在居家康复，如何购买日常生活用品？ |
|  | My family member has tested positive for COVID, what should I do? | 我的家人确诊了新冠，我应该怎么做？ |
|  | My PCR result is positive, should I go to the hospital? | 我的核酸检测结果是阳性， 我应该去医院吗？ |
|  | What are the complications that COVID-19 have? | COVID 有什么后遗症? |
|  | What about rapid tests? | 什么是快速检测？ |
|  | What are suspected cases and confirmed cases? | 什么是疑似病例和确诊病例？ |
|  | What are the case counts today? | 今天有多少确诊病例？ |
|  | What are the clinically available treatments for COVID-19? | 临床上新冠治疗的手段有哪些? |
|  | What are the different types of vaccines we can take in Singapore? | 新加坡有哪些不同品牌的新冠疫苗？ |
|  | What are the government policies regarding COVID-19 in Singapore? | 新加坡的防疫政策是怎样的？ |
|  | What are the ill effects of being infected with COVID-19? | 感染新冠病毒有什么后遗症？ |
|  | What are the ill effects of children being infected with COVID-19? | 儿童感染新冠病毒有什么后遗症？ |
|  | What are the ill effects of the vaccine? | 疫苗的副作用有哪些? |
|  | What are the latest safety measures? | 最新的安全管制措施？ |
|  | What are the long term ill effects of COVID-19? | 感染新冠病毒会有什么长期的后遗症？ |
|  | What are the requirements for returning to workplaces? | 回公司上班之前需要做什么？ |
|  | What are the side effects of the 3rd vaccination? | 第三支疫苗副作用有哪些？ |
|  | What are the symptoms of being infected with COVID-19? | 感染新冠病毒有什么症状？ |
|  | What are the symptoms of COVID-19? | COVID-19有什么症状？ |
|  | What are the symptoms that COVID-19 have? | COVID 的症状有哪些? |
|  | What are the variants of SARS-Cov-2 virus actively spreading in Singapore? | 新加坡现在有哪些新冠变异毒株？ |
|  | What do I need to take note of when on home recovery programme? | 居家康复时需要注意什么？ |
|  | What documents do I need to prepare before travelling under VTL? | 出国前需准备那些证件？ |
|  | What happens to people who get COVID-19? | 得了COVID-19的人会怎么样？ |
|  | What illnesses can an infected person sustain? | 感染上冠病会有什么疫病？ |
|  | What are Singapore's latest travel guidelines? | 新加坡最新的旅游政策? |
|  | What is the COVID-19 information hotline? | 关于新冠病毒信息的热线电话是多少？ |
|  | What is the cure rate of COVID-19? | 新冠的治愈率？ |
|  | What is the difference between COVID-19 and the common flu? | 冠状病毒和普通流感有何不同？ |
|  | What is the difference between Health Risk Warning and Health Risk Alert? | 健康风险警告和健康风险警戒有什么区别？ |
|  | What is the difference between isolation and quarantine? | 隔离和检疫有什么区别？ |
|  | What is the difference between COVID-19 pneumonia, flu and common cold? | 新型冠状病毒肺炎与流感、普通感冒有什么区别？ |
|  | What is the infection risk for children? | 儿童感染冠状病毒的几率有多大？ |
|  | What is the latest development of medications for COVID-19? | 新冠药物的最新情况？ |
|  | What is the limit on the number of visitors per day now? | 现在每天可以有多少访客？ |
|  | What is the mortality rate of COVID-19? | 新冠的死亡率是多少？ |
|  | What is the number of deaths from COVID-19 in Singapore? | 新加坡新冠病毒的病死率是多少？ |
|  | What is the percentage of people who suffer from long term effects after being infected with COVID-19? | 得了新冠以后有长期后遗症的比例是多少? |
|  | What is the possibility of me testing negative for COVID-19 when I am carrying the infection? | 冠状病毒患者检测呈阴性的几率有多大？ |
|  | What is the probability I will be infected by COVID-19? | 我感染冠状病毒的几率有多大？ |
|  | What is the standard to assess when COVID-19 ends? | 新冠病毒结束的标准是什么？ |
|  | What is the transmission rate of COVID-19? | 新冠病毒的传播率？ |
|  | What is the vaccination rate in Singapore currently? | 新加坡现在的疫苗覆盖率达到多少？ |
|  | What lifestyle practices will increase the risk of being infected? | 有什么生活习惯会导致人更容易受冠状病毒感染？ |
|  | What medications can treat COVID-19? | 有什么药物能治愈冠状病毒？ |
|  | What medications should I eat to prevent COVID-19? | 我该吃什么药预防？ |
|  | What should I do if I have been exposed to someone who has COVID-19? | 如果我与一位COVID-19患者有过接触，该怎么办？ |
|  | What should I do if I have been issued Home Quarantine Order? | 接到隔离令之后应该怎么做？ |
|  | What should I do if I have COVID-19 symptoms? | 如果我有COVID-19症状，我应该怎么做？ |
|  | What should I do if I received a Health Risk Warning? | 接到健康风险警戒应该怎么做？ |
|  | What should I do if my house is not suitable for home recovery? | 我家不适合居家康复怎么办？ |
|  | What should I take note of in my daily life? | 日常生活要注意什么? |
|  | What test should I get to see if I have COVID-19? | 我应该做什么检测来确定是否患COVID-19？ |
|  | What to do if the ART indicates positive? | 抗原检测阳性怎么办？ |
|  | What to do when I receive a health risk warning from MOH? | 接到卫生部的简讯该这么办？ |
|  | What is the process of home recovery? | 居家康复的流程是什么？ |
|  | What is the range of medical expenses for COVID-19 treatment? | 新冠的大约治疗费用? |
|  | What is the situation of the availability of medical resources in Singapore? | 新加坡医疗资源的占用情况? |
|  | What is the transmission rate of COVID-19 in Singapore now? | 现在新加坡新冠疫情的传播率是多少？ |
|  | When can I not wear a mask? | 我什么时候可以不戴口罩？ |
|  | When can medications that treat COVID-19 be mass produced? | 新冠病毒的特效药什么时候可以大量生产？ |
|  | When should I get a test for COVID-19? | 我应该什么时候做COVID-19检测？ |
|  | When will new and more effective vaccines be available? | 新的更有效的疫苗何时研发出来？ |
|  | When will the COVID-19 pandemic end? | 新冠病毒流行何时结束？ |
|  | Where can I be tested for COVID-19 infection? | 我在哪里可以做新冠核酸检查？ |
|  | Where can I buy ART kits? | 哪里可以买 ART kit? |
|  | Where can I purchase a rapid test kit? | 我在哪里可以买到快速抗原检测？ |
|  | Which clinics provide PCR testing? | 哪些诊所可以做核酸检测？ |
|  | Which countries can I go under the VTL? | 打了疫苗可以去那些国家旅游？ |
|  | Which COVID-19 vaccines are available? | 冠状病毒潜伏期多长？ |
|  | Which of the WHO-approved vaccines are the most effective? | 目前WHO批准的疫苗哪一款最有效？ |
|  | Which vaccines are the most effective? | 哪种疫苗最有效？ |
|  | Which kind of masks should I wear? | 我应该带什么类型的口罩？ |
|  | Who can take the third dose of vaccination? | 谁可以打第三支疫苗？ |
|  | Who is at high risk of COVID infection? | 冠状病毒高危群体包括哪些？ |
|  | Who is most at risk of severe illness from COVID-19? | 谁最有可能因COVID-19而患上严重疾病？ |
|  | Why did the government only allow vaccinated people to go to the mall? | 为什么政府只应许打了疫苗的人去商场？ |
|  | Will all close contacts need to be tested for COVID-19? | 所有的密切接触者需要做冠病检测吗？ |
|  | Will COVID-19 last forever for humans? | 新冠病毒是否跟人类永远公存? |
|  | Will we have to take a booster vaccination every 6 months moving forward? | 疫苗会变成每半年需打的现象吗？ |
|  | With the evolution of COVID variants, what does this mean for us? | 冠状病毒变异，意味着什么？ |
| **Malay** | Am I needed to quarantine if a household member is a close contact with a positive case? | Adakah saya perlu jalani kuarantin sekiranya ada ahli rumah yang dikuarantin disebabkan mempunyai kontak rapat dengan pesakit positif COVID-19? |
|  | Am I needed to take a COVID-19 test after returning from abroad? | Adakah saya perlu menjalankan ujian COVID-19 jika saya baru pulang dari luar negara? |
|  | Are all children suitable for vaccination? | Adakah vaksin sesusai untuk semua kanak-kanak? |
|  | Are babies immune to COVID-19? | Adakah bayi kebal daripada COVID-19? |
|  | Are cloth masks effective in protecting you against COVID-19? | Adakah menggunakan kain pelitup muka berkesan mencegah dijangkitti COVID-19? |
|  | Are cloth masks safe to protect against COVID-19? | Adakah pelitup kain dapat mecegah daripada COVID-19? |
|  | Are face coverings mandatory while exercising? | Adakah pelitup muka diwajibkan semasa bersenam? |
|  | Are kids younger than 7 immune from COVID-19? | Adakah kanak-kanak di bawah umur 7 tahun kebal daripada COVID-19? |
|  | Are COVID-19 vaccines safe for human beings? | Adakah vaksin COVID-19 selamat untuk manusia? |
|  | Are the test kits different for Omicron variant? | Adakah kit ujian berbeza untuk varian Omicron? |
|  | Are there any age restrictions on the use of Antigen Rapid Test (ART) kits? | Adakah terdapat sebarang had umur terhadap penggunaan kit Ujian Pantas Antigen (ART)? |
|  | Are there any alternatives to vaccination? | Adakah terdapat sebarang alternatif untuk vaksinasi? |
|  | Are there any risks in getting vaccinated from COVID-19? | Adakah terdapat sebarang risiko dalam mendapatkan vaksin COVID-19? |
|  | Are we going to receive vaccine every year in the future? | Adakah kita akan menerima vaksin setiap tahun pada masa akan datang? |
|  | At what age can a person take a COVID-19 vaccine? | Umur berapakah boleh seseorang mengambil vaksin COVID-19? |
|  | Between wearing a face mask or N-95, which helps to reduce COVID-19 transmission? | Sekiranya saya memakai pelitup muka atau N-95, adakah ianya akan membantu untuk mengurangkan penularan COVID-19? |
|  | By which date must I get my booster shot? | Bila tarikh yang paling lewat saya seharusnya mengambil vaksinasi booster? |
|  | Can COVID-19 cause death? | Adakah penyakit COVID-19 ini boleh menyebabkan kematian? |
|  | Can COVID-19 spread through the water reservoir? | Bolehkah COVID-19 merebak melalui takungan air? |
|  | Can I care for someone with COVID-19 safely? | Bolehkah saya menjaga seseorang yang menghidap COVID-19 dengan selamat? |
|  | Can I choose to get different vaccines for the first and second dose? | Bolehkah saya pilih vaksin yang berlainan untuk dos pertama dan kedua? |
|  | Can I get infected by shaking hands of someone who is contracted with this virus? | Bolehkan saya dapat jangkitan virus selepas bersalaman dengan seseorang yang mendapat virus ini? |
|  | Can I share food and drinks with family members who have recovered from COVID-19? | Bolehkah saya berkongsi makanan dan minuman dengan ahli keluarga yang telah pulih daripada COVID-19? |
|  | Can I still be infected with COVID-19 if I had already contracted it? | Bolehkah saya masih dijangkiti COVID-19 jika saya telah dijangkiti? |
|  | Can I take another vaccine at the same time I take the COVID-19 vaccine? | Bolehkah saya mengambil vaksin lain pada masa yang sama saya mengambil vaksin COVID-19? |
|  | Can I take COVID-19 vaccines if I have drug allergies? | Bolehkah saya mengambil vaksin COVID-19 jika saya ada alahan ubat? |
|  | Can I use face shield instead of a face mask if I have pre-existing conditions such as asthma? | Bolehkah saya menggunakan pelindung muka dan bukannya pelitup muka jika saya mempunyai penyakit seperti asma? |
|  | Can someone spread the virus even when he wears a mask? | Bolehkah seseorang menular virus ini walaupun dia memakai pelitup? |
|  | Can COVID-19 disease cause long-term effects? | Adakah penyakit COVID-19 ini boleh menyebabkan kesan jangka panjang? |
|  | Children are not vaccinated. How can I protect them against COVID-19? | Kanak-kanak tidak di vaksin. Apakah yang boleh dilakukan untuk mencegah dari COVID-19? |
|  | Do I have to pay for visits to the doctor for any vaccine-related injuries? | Adakah saya perlu membayar untuk lawatan ke doktor untuk sebarang kecederaan berkaitan vaksin? |
|  | Do I have to take a fourth booster shot? | Perlukah saya mengambil booster vaksinasi keempat? |
|  | Do I have to undergo quarantine if any of my household members are close contacts of COVID-19 positive patients? | Adakah saya perlu menjalani kuarantin sekiranya ada ahli keluarga rumah yang dikuarantin disebabkan mempunyai kontak rapat dengan pesakit COVID-19? |
|  | Do I have to wear a mask while exercising or at leisure? | Adakah pelitup muka diwajibkan ketika bersenam atau beriadah? |
|  | Do you think there will be an end to COVID-19? | Adakah anda rasa COVID-19 akan berakhir? |
|  | Does current vaccine or booster protect against Omicron variant? | Adakah vaksin/ vaksin booster berkesan mencegah dijangkitti COVID-19? |
|  | How can I ensure my children are protected from COVID-19 if they cannot be vaccinated? | Bagaimanakah saya boleh memastikan anak-anak saya dilindungi daripada COVID-19 jika mereka tidak boleh diberi vaksin? |
|  | How can I get a COVID-19 vaccine? | Bagaimana saya boleh mendapat vaksin COVID-19? |
|  | How can I get the latest COVID-19 news updates? | Bagaimanakah Saya boleh mendapatkan maklumat terkini berkaitan penyakit COVID-19? |
|  | How can I report if I start to feel side effects of the COVID-19 vaccine? | Bagainmana cara saya membuat lapuran jika berlaknya kesan sampingan vaskin COVID-19? |
|  | How did COVID-19 spread? | Bagaimanakah COVID-19 merebak? |
|  | How do I enter quarantine if I am infected with COVID-19? | Kalau saya mengdapa COVID-19, bagaimana saya masuk ke kuarantin? |
|  | How do I treat COVID-19 vaccine side effects? | Bagaimanakah cara saya merawat kesan sampingan vaksin COVID-19? |
|  | How do we prevent infection of COVID-19? | Apakah langkah-langkah pencegahan jangkitan COVID-19? |
|  | How do you check for negative, positive or invalid test result from the ART test? | Bagaimanakah anda menyemak keputusan negatif, positif atau tidak sah daripada alat ujian ART? |
|  | How does MOH determine if someone is a close contact of a confirmed case? | Bagaimanakah MOH menentukan sama ada seseorang itu adalah kenalan rapat kes yang disahkan? |
|  | How does the dosage of vaccine differ between adults and children? | Bagaimanakah dos vaksin berbeza antara orang dewasa dan kanak-kanak? |
|  | How does the vaccine work? | Bagaimana vaksin berfungsi? |
|  | How effective is alcohol hand rub against the COVID-19 virus? | Adakah menggunakan sapu tangan alkohol berkesan mencegah dijangkitti COVID? |
|  | How effective is the booster jab? | Adakah vaksin booster berkesan? |
|  | How effective is vaccination? | Sejauh manakah vaksinasi berkesan? |
|  | How effective is wearing a mask to prevent COVID-19? | Adakah menggunakan pelitup muka berkesan mencegah dijangkitti COVID-19? |
|  | How is contact tracing being conducted? | Bagaimanakah pengesanan kontak dilakukan? |
|  | How is COVID-19 different from normal flu? | Apa perbezaan COVID-19 dan penyakit selsema biasa? |
|  | How long is Health Risk Warning? | Berapa lama Perintah Amaran Risiko Kesihatan? |
|  | How long should someone isolate if they are COVID-19 positive? | Berapa lama seseorang harus mengasingkan diri jika mereka positif COVID-19? |
|  | How many days after exposure do symptoms appear? | Berapa hari kah tanda-tanda jangkitan muncul selepas terdedah dengan virus ini? |
|  | How much do I pay if I go to a PHPC clinic for respiratory symptoms? | Berapakah bayaran jika saya ke klinik PHPC untuk simptom jankitan pernafasan? |
|  | How should I protect myself against COVID-19? | Bagaimanakah saya boleh melindungi diri saya? |
|  | How to use Antigen Rapid Test (ART) self test kit? | Bagaimana untuk mengambil ujian pantas antigen (ART)? |
|  | I have only cough and flu symptoms. Do I need to go to the clinic to take an ART test? | Saya mempunyai gejala batuk dan selesema sahaja. Perlukah saya ke klinik untuk mengambil ujian saringan? |
|  | I live with somebody who has tested positive for COVID-19 whereas I have tested negative, what should I do? | Saya tinggal bersama seorang yang telah diuji positif untuk COVID-19 sedangkan saya telah mendapat ujian negatif, apakah yang perlu saya lakukan? |
|  | I received a Health Risk Warning (HRW) via SMS. Can I claim compensation for the time lost at work due to self-isolation? | Saya menerima Amaran Risiko Kesihatan (HRW) melalui SMS. Bolehkah saya menuntut pampasan untuk masa yang ketiadaan saya di tempat kerja akibat pengasingan diri? |
|  | I was registered as a household contact of a confirmed case, but have not received a Health Risk Warning (HRW) even after 48 hours. What should I do? | Saya telah didaftarkan sebagai kenalan isi rumah bagi kes yang disahkan, tetapi tidak menerima Amaran Risiko Kesihatan (HRW) walaupun selepas 48 jam. Apa patut saya buat? |
|  | If ART test is positive, do I need to do a PCR test? | Kalau saya ujian ART positif, adakah saya perlu ulangkan PCR test? |
|  | If I am COVID recovered, will I get COVID-19 again? | Kalau saya sudah sembuh dari COVID, bolehkah saya menjangkiti COVID-19 lagi? |
|  | If I am pregnant, can I take the COVID-19 vaccine? | Jika saya hamil, bolehkah saya mengambil vaksin COVID-19? |
|  | If I breastfeed my baby, can I take the COVID-19 vaccine? | Jika saya menyusukan bayi, bolehkah saya mengambil vaksin COVID-19? |
|  | If I have a rash after the COVID-19 vaccine, what should I do? | Jika saya mengalami ruam selepas vaksin COVID-19, apa patut saya lakukan? |
|  | If I have been infected with COVID-19, should I take the vaccine? | Jika saya pernah dijangkiti COVID-19, perlukah saya mengambil vaksin ini? |
|  | If I have COVID-19 symptoms, what should I do? | Jika saya ada simptom COVID-19, apa patut saya lakukan? |
|  | If I have ever been infected with COVID-19, do I still need to get the vaccine? | Jika saya pernah dijangkiti COVID-19, adakah saya masih perlu mendapatkan vaksin? |
|  | If I have information regarding a breach in regulations, what is the appropriate channel to report? | Jika saya mempunyai maklumat mengenai pelanggaran SOP, bagaimanakah saya boleh melaporkannya? |
|  | If I was COVID positive, can I still receive vaccine or booster? | Kalau saya positif COVID, bolehkah saya menerima vaksin? |
|  | Is COVID-19 dangerous? | Adakah COVID-19 bahaya? |
|  | Is COVID-19 dangerous? | Adakah COVID-19 bahaya? |
|  | Is COVID-19 less lethal if you are younger? | Adakah COVID-19 kurang membawa maut jika anda lebih muda? |
|  | Is double masking effective against COVID virus? | Adakah menggunakan dua keping pelitup muka berkesan mencegah dijangkitti COVID? |
|  | Is eye protection necessary? | Adakah perlindungan mata diperlukan? |
|  | Is frequent Antigen Rapid Test (ART) swabbing harmful to me? | Adakah menggunakan Antigen Rapid Test (ART) yang kerap berbahaya kepada saya? |
|  | Is home recovery safe? | Adakah pemulihan di rumah selamat? |
|  | Is it important to ensure my child is vaccinated? | Pentingkah kalau anak saya divaksinasi? |
|  | Is it necessary to wear N95 mask in public? | Perlukah memakai pelitup N95 di tempat awam? |
|  | Is it safe to be vaccinated from COVID-19? | Adakah vaksinasi untuk COVID-19 selamat? |
|  | Is it true that the procedure of swab test is painful and the pain reaches the brain? | Betul Ke Prosedur 'Swab Test' Rasa Sakitnya Sampai Ke Otak? |
|  | Is mixing different brands of vaccine and booster safe for everyone? | Adakah mencampurkan vaksin dengan vaksin booster jenama berbeza selamat untuk semua orang? |
|  | Is N-95 necessary? | Adakah N-95 diperlukan? |
|  | Is the COVID-19 vaccine safe for children? | Adakah vaksinasi COVID-19 selamat untuk golongan-golongan warga emas kanak-kanak? |
|  | Is the COVID-19 vaccine safe for me if I have drug/ food allergies? | Adakah vaksinasi COVID-19 selamat jika saya ada alahan ubat/makanan? |
|  | Is the COVID-19 vaccine safe for patients with chronic diseases? | Adakah vaksinasi COVID-19 selamat untuk golongan-golongan warga emas penghidap penyakit kronik? |
|  | Is the COVID-19 vaccine safe for the elderly? | Adakah vaksinasi COVID-19 selamat untuk golongan-golongan warga emas? |
|  | Is the COVID-19 vaccine safe? | Adakah vaksin COVID-19 selamat? |
|  | Is the Omicron variant dangerous? | Adakah variant Omicron itu bahaya? |
|  | Is the vaccine dangerous? | Apakah bahaya untuk vaksin COVID? |
|  | Is the vaccine effective for protection against this virus? | Adakah vaksin berkesan untuk melindungi daripada virus ini? |
|  | Is the vaccine safe for children? | Adakah vaksin selamat untuk kanak-kanak? |
|  | Is there any remedy for COVID-19? | Apakah rawatan untuk penyakit COVID-19? |
|  | I've been to an affected area, but did not receive a Health Risk Warning (HRW) notification. Should I be concerned? | Saya pernah ke kawasan yang berisiko tinggi, tetapi tidak menerima pemberitahuan Amaran Risiko Kesihatan (HRW). Patutkah saya risau? |
|  | Must I wear a mask throughout the day at work/ school, if there is a distance of more than 1 metre between my seat and the other person’s? | Perlukah saya memakai pelitup muka sepanjang hari di tempat kerja/ sekolah, jika terdapat jarak lebih daripada 1 meter antara tempat duduk saya dengan orang lain? |
|  | My colleague/ classmate/ friend is travelling to a country with COVID-19 cases. When they return, do they need to isolate themselves even if they test negative? | Rakan sekerja/ rakan sekelas/ rakan saya sedang melancong ke negara yang mempunyai kes COVID-19. Apabila mereka kembali, adakah mereka perlu mengasingkan diri walaupun negatif? |
|  | Should I get the booster dose? | Haruskah saya mendapatkan suntikan dos penggalak? |
|  | Should I let my child continue to play sports with friends? | Bolehkah saya membiarkan anak saya terus bermain sukan dengan rakan-rakan? |
|  | Should I take the booster vaccine if I had COVID-19 prior? | Perlukah saya mengambil suntikan vaksin booster jika saya pernah dijangkiti COVID-19? |
|  | Should I use self-test kit even when I do not have any symptoms? | Bolehkah saya gunakan kit ujian sendiri walaupun tiada sebarang gejala? |
|  | Should I wear gloves when going out? | Perlukah saya memakai sarung tangan semasa keluar? |
|  | What advice can I follow as a healthy individual? | Apakah nasihat untuk saya yang masih sihat? |
|  | What am I allowed to do while on Home Quarantine? | Apakah yang perlu dilakukan ketika di bawah perintah pengawasan dan pemerhatian di rumah? |
|  | What are some measures that government should impose to prevent Omicron clusters from happening? | Apakah langkah-langkah yang perlu dikenakan oleh kerajaan untuk mengelakkan gugusan Omicron daripada berlaku? |
|  | What are some of the side effects that can occur because of the vaccine? | Apakah kesan sampingan yang boleh berlaku dengan vaskin COVID-19? |
|  | What are some ways citizens can do to help the fight against COVID-19? | Apakah beberapa cara yang boleh dilakukan oleh rakyat untuk membantu menentang COVID-19? |
|  | What are symptoms of COVID-19? | Apakah tanda-tanda jangkitan COVID-19? |
|  | What are the chances of getting COVID-19 if I am not vaccinated? | Jika saya tidak divaksin,adakah saya masih boleh dijangkiti COVID-19? |
|  | What are the common side effects from the COVID vaccines? | Apakah kesan-kesan daripada vaksin COVID-19? |
|  | What are the complications after you have recovered from COVID-19? | Apa komplikasi setelah sembuh dari COVID-19? |
|  | What are the COVID-19 symptoms? | Apakah simptom COVID-19? |
|  | What are the differences between COVID-19 and SARS? | Apa perbezaannya COVID-19 dengan SARS? |
|  | What are the differences in COVID-19 vaccines? | Apakah perbezaan dalam vaksin COVID-19? |
|  | What are the different kinds of mask? | Apakah jenis jenis topeng yang berbeza? |
|  | What are the different tests for COVID-19? | Apakah jenis ujian COVID-19 yang dijalankan? |
|  | What are the effects of vaccination on COVID-19 patients? | Adakah kesan pengambilan vaksin terhadap pesakit COVID-19? |
|  | What are the high risk groups? | Siapakah golongan berisiko tinggi? |
|  | What are the ingredients inside the COVID-19 vaccine? | Apakah bahan- bahan yang terkandung dalam vaksin COVID-19 |
|  | What are the measures taken to prevent the spread of COVID-19? | Apakah langkah-langkah pencegahan jangkitan COVID-19? |
|  | What is the next step to be done if I am confirmed positive with COVID-19? | Apakah tindakan selanjutnya sekiranya keputusan ujian disahkan positif? |
|  | What are the next steps to be taken if I am positive on my COVID-19 test? | Apakah tindakan selanjutnya sekiranya keputusan ujian COVID 19 disahkan positif? |
|  | What are the possible treatment options for COVID-19? | Apakah rawatan untuk penyakit COVID-19? |
|  | What are the procedures to follow when returning from abroad? | Apakah prosedur yang perlu dilakukan apabila pulang dari luar negara? |
|  | What are the requirements needed to be able to get vaccinated? | Apakah keperluan yang diperlukan untuk mendapatkan vaksin? |
|  | What are the short term effects of COVID-19? | Apakah kesan jangka pendek COVID-19? |
|  | What are the side effects of COVID-19 vaccine? | Apakah kesan sampingan vaksinasi COVID-19? |
|  | What are the signs and symptoms of COVID-19? | Apakah tanda-tanda COVID-19? |
|  | What are the signs and symptoms to look out for in COVID-19 patients? | Apakah kriteria saya disyaki menghidap penyakit COVID-19? |
|  | What are the steps that I can follow if I am suspected to be COVID positive? | Apakah tindakan yang harus dilakukan jika saya disyaki menghidap COVID-19? |
|  | What are the steps to detect COVID-19? | Apakah langkah-langkah untuk mengesan COVID-19? |
|  | What are the type of vaccines for COVID-19? | Apakah jenis Vaksinasi untuk COVID-19? |
|  | What are the types of COVID-19 testing? | Apakah jenis ujian COVID-19 yang dijalankan? |
|  | What are the ways I can lessen the side effects? | Bagaimanakah saya dapat merawat kesan sampingan vaskin COVID-19? |
|  | What can I do if I live alone and am COVID positive? | Apakah patut saya buat kalau saya tinggal keseorangan dan ada COVID? |
|  | What do I do if have side effects from vaccine? | Apakah yang perlu saya lakukan jika mempunyai kesan sampingan daripada vaksin? |
|  | What do I have to do in order to avoid getting COVID-19 in shopping centres? | Apakah langkah pencegahan jangkitan COVID-19 di pusat beli belah? |
|  | What does it mean by close contact? | Apakah yang dimaksudkan dengan kontak rapat? |
|  | What happens if my family member tested positive? | Apak yang berlaku jika keluarga saya diuji positif? |
|  | What if I am allergic to hand sanitiser? | Bagaimana jika saya alah kepada pembersih tangan? |
|  | What if I am not eligible for vaccine? | Bagaimana jika saya tidak layak mendapat vaksin? |
|  | What is COVID? | Apa itu COVID? |
|  | What is meant by a close contact? | Apakah yang dimaksudkan dengan kontak rapat? |
|  | What are the criteria to suspect for COVID-19? | Apakah Kriteria saya disyaki menghidap penyakit COVID-19? |
|  | What is the difference between ART and PCR? | Apakah perbezaan antara ART dan PCR? |
|  | What is the difference between Omicron and Delta? | Apakah perbezaan varian Omicron dan Delta? |
|  | What is the difference between Pfizer and Moderna vaccines? | Apakah perbezaan di antara vaksin Pfizer dan Moderna? |
|  | What is the difference between Pfizer, Moderna and Sinopharm? | Apakah perbezaan vaksin Pfizer, Moderna dan Sinopharm? |
|  | What is the difference between the variants of COVID-19 in terms of symptoms? | Apakah perbezaan antara varian COVID-19 dari segi gejala/simptom? |
|  | What is the ideal time distance to take another vaccine for the COVID-19 vaccine? | Apakah jarak masa yang sesuai untuk mengambil vaksin lain selapas vaksin COVID-19? |
|  | What is the meaning of COVID-19? | Erti COVID-19? |
|  | What is the PHPC Clinic? | Apakah PHPC clinic? |
|  | What is the rate of transmission among different variants of COVID-19? | Apakah kadar penularan antara varian COVID-19 yang berbeza? |
|  | What is the side effect of COVID-19 vaccine? | Apakah kesan sampingan vaksin COVID-19? |
|  | What is the side effect of the COVID-19 vaccine? | Apakah kesan sampingan vaksin COVID-19? |
|  | What is the strategy to reduce the number of COVID-19 cases? | Apakah strategi bagi mengurangkan jumlah kes COVID-19 |
|  | What is the treatment for COVID-19 virus? | Apakah rawatan jangkitan virus COVID-19? |
|  | What is the treatment for COVID-19? | Apakah rawatan untuk COVID-19? |
|  | What rules should I follow if I get COVID-19? | Apakah peraturan yang perlu saya patuhi jika saya mendapat COVID-19? |
|  | What should be done when I am under SHN notice, if I test positive? | Apakah yang perlu dilakukan apabila saya di bawah notis SHN, jika saya positif? |
|  | What should I do after knowing my PCR result is positive? | Apa yang harus saya lakukan jikalau PCR saya positif? |
|  | What should I do after knowing my self-test kit result is positive? | Apakah harus saya lakukan setelah mengetahui yang keputusan ujian sendiri adalah positif? |
|  | What should I do if I am having signs and symptoms of COVID-19? | Apakah tindakan yang patut saya ambil jika saya menghidapi tanda-tanda COVID-19? |
|  | What should I do if I have symptoms of COVID-19? | Apakah yang saya harus lakukan jikalau alami tanda-tanda jangkitan COVID-19? |
|  | What should I do if my doctor advises me not to get vaccinated? | Apakah yang perlu saya lakukan sekiranya doktor menasihatkan saya supaya tidak diberi vaksin? |
|  | What should you avoid doing after vaccination? | Apakah yang perlu anda elakkan selepas vaksinasi? |
|  | When can I get my booster shot? | Bilakah saya boleh menambil suntikan penggalak? |
|  | When do I need a booster? | Bilakah saya perlu mengambil booster? |
|  | When should I see a doctor? | Bila patut saya perlu berjumpa doktor? |
|  | When should I seek emergency help? What symptoms should I look out for? | Bilakah saya perlu mendapatkan bantuan kecemasan? Apakah gejala yang perlu saya perhatikan? |
|  | When should I seek treatment at the Emergency Department? | Bilakah saya patut ke Kecemasan untuk rawatan? |
|  | When should I use self-test kit? | Bilakah harus saya lakukan kit ujian sendiri? |
|  | When should you use the ART test kit? | Bilakah anda perlu menggunakan alat ujian ART? |
|  | When will I receive my PCR test results? | Bila akan saya dapat keputusan ujian PCR saya? |
|  | Where can I get mask from? | Dari mana saya boleh mendapatkan topeng? |
|  | Where can I get the latest information regarding COVID-19? | Bagaimanakah saya boleh mendapatkan maklumat terkini tentang COVID-19? |
|  | Where can I get vaccinated? | Di mana boleh saya mendapatkan suntikan vaksin? |
|  | Where can you get ART test kit? | Di mana anda boleh mendapatkan alat ujian ART? |
|  | Where do I get the latest updates for COVID-19? | Bagaimanakah saya boleh mendapatkan maklumat terkini berkaitan penyakit COVID-19? |
|  | Which groups are at risk of COVID-19? | Siapakah golongon berisiko tinggi? |
|  | Which is riskier; getting infected by COVID-19 or getting vaccinated from COVID-19? | Mana yang lebih berisiko; dijangkiti COVID-19 atau divaksin daripada COVID-19? |
|  | Which phone number should I contact after knowing my self-ART result is positive? | Apakah nombor talian yang saya harus saya kunjung selepas mengetahui yang keputusan ART sendirian saya positif? |
|  | Which test is more efficient in detecting COVID-19? | Ujian yang manakah lebih cekap mengesan COVID-19? |
|  | Which vaccines are approved by the government? | Apakah vaksin yang diluluskan oleh pemerintah Singapura? |
|  | Who are the high risk individuals? | Siapakah golongan yang berisiko tinggi? |
|  | Who can receive the vaccine? | Siapa yang mendapatkan vaksin? |
|  | Who is suitable to be treated in the CTF? | Siapakah yang sesuai mendapatkan rawatan di CTF? |
|  | Who will receive the "Health Risk Warning"? | Siapa yang akan menerima "Health Risk Warning"? |
|  | Who would be exempted from taking the vaccine? | Siapa yang tidak seharusnya mendapatkan vaskin? |
|  | Why am I required to take boosters? | Mengapakah saya perlukan vaksin booster? |
|  | Why do I have to quarantine if I am infected with COVID-19? | Mengapakah saya perlu dikuarantin jikalau dijangkiti COVID-19? |
|  | Why do I need a booster? | Kenapa saya perlu mengambil booster? |
|  | Why do we need to receive booster jabs? | Mengapakah kita perlu menerima vaksin booster? |
|  | Why is COVID 19 so easily transmissible? | Mengapakah COVID 19 begitu mudah dijangkitkan? |
|  | Will children experience the similar side effect as adult after receiving vaccine? | Apakah kanak-kanak yang menerima vaksin akan mengalami kesan sampingan sama seperti orang dewasa? |
|  | Will COVID-19 hurt my natural immunity? | Adakah COVID-19 akan menjejaskan imuniti semula jadi saya? |
|  | Will COVID-19 vaccines and booster jabs lower my natural immunity? | Adakah vaksin COVID-19 dan suntikan penggalak akan mengurangkan imuniti semula jadi saya? |
| **Tamil** | Can a patient post-bypass surgery be vaccinated? | போஸ்ட் பைபாஸ் அறுவை சிகிச்சை நோயாளிக்கு தடுப்பூசி போட முடியுமா? |
|  | Can I get COVID from my pets or other animals? | எனது செல்லப்பிராணிகளிடமிருந்தோ அல்லது மற்ற விலங்குகளிடமிருந்தோ நான் கோவிட் பெறலாமா? |
|  | Does COVID vaccination affect the menstrual cycle? | கோவிட் தடுப்பூசி மாதவிடாய் சுழற்சியை பாதிக்கிறதா? |
|  | Does extreme tiredness occur due to COVID? | தீவிர சோர்வுக்கு கோவிட் காரணமா? |
|  | How can prevent myself from getting COVID? | கோவிட் நோயிலிருந்து என்னை நான் எவ்வாறு தடுப்பது? |
|  | How do I overcome stress and depression during the pandemic? | தொற்றுநோய்களின் போது மன அழுத்தம் மற்றும் மனச்சோர்வை எவ்வாறு சமாளிப்பது? |
|  | How do I report COVID-19 vaccine side effects? | கோவிட் -19 தடுப்பூசி பக்க விளைவுகளை நான் எவ்வாறு தெரிவிப்பது? |
|  | How many days will it take to cure COVID for a person without any comorbidities? | எந்த நோய்களும் இல்லாத ஒருவருக்கு கோவிட் குணப்படுத்த எத்தனை நாட்கள் ஆகும் ? |
|  | How to take care of children with COVID? | குழந்தைகளுக்கு கோவிட் பாதிப்பு ஏற்படும் போது எப்படி கவனிப்பது? |
|  | If I have skin allergy or problems, can I use hand sanitizers? Are there any specific hand sanitizers for skin problems or allergies? | எனக்கு தோல் ஒவ்வாமை அல்லது பிரச்சனை இருந்தால் சானிடைசரைப் பயன்படுத்தலாமா? தோல் பிரச்சனைகள் அல்லது ஒவ்வாமைகளுக்கு ஏதேனும் குறிப்பிட்ட சானிடைசர் இருந்தால்? |
|  | If two people tested positive, can they quarantine together or do they have to be separated ? | இரண்டு பேருக்கு நேர்மறை சோதனை நடந்தால், அவர்கள் ஒன்றாக தனிமைப்படுத்த முடியுமா அல்லது அவர்கள் பிரிக்கப்பட வேண்டுமா? |
|  | Is the rate of heart attack higher after vaccination? | தடுப்பூசி போட்ட பிறகு மாரடைப்பு விகிதம் அதிகமாக உள்ளதா? |
|  | What complications are possible after COVID? | கோவிட் பிறகு என்ன சிக்கல்கள் சாத்தியம் ? |
|  | What should I do if I get mild symptoms? | லேசான அறிகுறிகள் தென்பட்டால் நான் என்ன செய்ய வேண்டும்? |
|  | Which COVID vaccine is very effective for the older age group? | எந்த கோவிட் தடுப்பூசி மிகவும் பயனுள்ளதாக இருக்கிறது மற்றும் வயதானவர்கள் எடுக்கலாம்? |
|  | Which vaccine is best for COVID, Covishield or Covaxin? | எந்த தடுப்பூசி சிறந்த, Covisheild அல்லது Covaxin? |
|  | Will children get infected from family members? | குடும்ப உறுப்பினர்களால் குழந்தைகள் பாதிக்கப்படுவார்களா? |
|  | Will I still be infected with COVID after vaccination? | தடுப்பூசி போட்ட பிறகும் நான் COVID ஆல் பாதிக்கப்படுவேனா? |
|  | Will there be any fluctuations in the COVID cases in the future? | கோவிட் பிந்தைய காலத்தில் மதிப்பில் அதிக ஏற்ற இறக்கங்கள் இருக்குமா ? |
|  | Will there be any memory loss as a post-COVID effect? | பிந்தைய கோவிட் விளைவு என ஏதேனும் நினைவக இழப்பு பிரச்சனை இருக்குமா? |
| **Filipino** | Can humans get COVID-19 from animals? | Maari ka bang mahawa ng COVID-19 mula sa hayop? |
|  | Can people without symptoms transmit the virus? | Maari bang maipasa ng taong may impeksyon ng COVID-19 kahit wala itong sintomas? |
|  | Can you catch COVID-19 from food? | Maari bang makuha ang COVID-19 sa pamamagitan ng pagkain? |
|  | Can you get infected twice with COVID-19? | Maaari ka pa bang mahawa ng mahigit isang beses ng COVID-19? |
|  | Can you have COVID-19 without symptoms? | Pwede ka bang magkaroon ng COVID-19 ng walang sintomas? |
|  | Can you still catch COVID-19 even if you are fully vaccinated? | Maaari ka pa bang makakuha ng COVID-19 kapag kumpleto na ang iyong bakuna? |
|  | How does using face mask prevent the spread of COVID-19? | Paano nakakatulong ang paggamit ng maskara sa mukha na labanan ang pagkalat ng COVID-19? |
|  | How is COVID transmitted? | Paano naipapasa ang COVID? |
|  | How soon before symptoms start can someone infected with COVID-19 be contagious? | Kailan maaaring magsimulang makahawa ang COVID-19 makaraan lumabas ang sintomas nito? |
|  | Is COVID-19 going to become seasonal? | Maari bang maging pana-panahon ang COVID-19? |
|  | Is hand sanitizer effective against COVID-19? | Epektibo ba ang paggamit ng saniteryer ng kamay laban sa COVID-19? |
|  | Should I still get my COVID-19 jab even if I have recovered from the virus? | Kailangan ko pa bang magpabakuna laban sa COVID-19 kapag nagkaroon na ko nito? |
|  | What are possible side effects of the COVID-19 vaccine? | Ano ang mga maaring maging masamang epekto ng bakuna laban sa COVID-19? |
|  | What are the symptoms of COVID? | Ano ang mga sintomas ng COVID? |
|  | What are the tests available to diagnose COVID-19? | Ano ang mga pasusuri na mayroon para malaman kung ako ay may COVID-19? |
|  | What is long COVID? | Ano ang mahabang COVID? |
|  | What is the benefit of getting the vaccine? | Ano ang benepisyo ng pagbakuna laban sa COVID-19? |
|  | What is the best way to avoid the spread of COVID-19? | Ano ang pinakamabisang paraan para maiwasan ang pagkalat ng COVID-19? |
|  | What should I do if I have been in contact with someone who has COVID-19 but I have no symptoms? | Ano ang kailangang kong gawin kung ako ay na lantad sa taong may sakit na COVID-19 at wala akong nararamdaman? |
|  | Where did COVID-19 originate? | Saan nagmula ang COVID-19? |
| **Thai** | Are there any herbal or alternative medicine for COVID-19? | มีการรักษาทางเลือกที่ไม่ใช้ยา หรือสมุนไพร สำหรับคนเป็นโรคโควิดไหม |
|  | Does each COVID-19 patient need hospital admission? | ผู้ป่วยโควิด ต้องนอนโรงพยาบาลทุกคนหรือเปล่า |
|  | How would COVID-19 infection spread? | โรคโควิดแพร่กระจายเชื้ออย่างไร |
|  | How long could COVID-19 survive on the surface? | เชื้อโควิดจะอยู่บนพื้นผิวได้นานเท่าไหร่ |
|  | How to protect ourselves from COVID-19 infection? | เราจะป้องกันโรคโควิดได้อย่างไร |
|  | How to treat COVID-19? | โรคโควิดมีวิธีการรักษาอย่างไร |
|  | If the self-testing kit for COVID-19 shows positive result, what should be the next step? | ถ้าผลตรวจโควิดที่ตรวจเองเป็นบวก ควรทำอย่างไรต่อ |
|  | Is there any activity I should not do on the day I get COVID-19 vaccination? | มีข้อห้ามอะไรที่ไม่ควรทำไหม ในวันที่ไปฉีดวัคซีนโควิด |
|  | Should everyone get vaccinated for COVID-19? | ทุกคนควรฉีดวัคซีนโควิดไหม |
|  | Should I buy a self-testing kit for COVID-19? | ควรซื้อชุดตรวจโควิด มาตรวจเองไหม |
|  | What are early symptoms of COVID-19? | อาการแรกเริ่มของคนที่ติดเชื้อโควิดคืออะไร |
|  | What are the complications of COVID-19 vaccination? | ภาวะแทรกซ้อนของวัคซีนโควิดคืออะไรบ้าง |
|  | What are the severe symptoms of COVID-19 patients? | อาการรุนแรงของผู้ป่วยโควิด คืออะไร |
|  | What is the delta variant of COVID-19? | โควิดสายพันธุ์เดลต้าคืออะไร |
|  | What is the mortality rate of COVID-19 patients? | ผู้ป่วยโควิด มีโอกาสตายมากน้อยแค่ไหน |
|  | What pathogen causes COVID-19? | โควิดเกิดจากเชื้ออะไร |
|  | When should pregnant woman get vaccinated for COVID-19? | ถ้ากำลังตั้งครรภ์ ควรฉีดวัคซีนโควิดเมื่อไหร่ |
|  | When will the COVID-19 pandemic end? | เมื่อไหร่การแพร่ระบาทโรคโควิดจะจบลง |
|  | Which COVID-19 vaccine has the best efficacy? | วัคซีนโควิดชนิดไหนมีประสิทธิภาพสูงสุด |
|  | Who should be tested for COVID-19 infection? | ใครบ้างที่ควรไปตรวจโควิด |
| **Japanese** | Are there any foods that are effective against the virus? | ウイルスに効果がある食品はありますか？ |
|  | Are there any preventative medicines? | 予防的な薬はありますか？ |
|  | Can I smoke? | タバコは吸っても良いですか？ |
|  | Do I need a mask if I get a vaccine? | ワクチンをすればマスクは不要ですか？ |
|  | Do young people get worse? | 若い人は重症化しないのですか？ |
|  | Does the effect vary depending on the type of vaccine? | ワクチンの種類によって効果は変わりますか？ |
|  | How effective is the vaccine? | ワクチンはどれくらい有効ですか？ |
|  | How long will the vaccine last? | ワクチンの効果はどれくらい続きますか？ |
|  | How well have the vaccines been developed? | 特効薬はどの程度開発されていますか？ |
|  | I don't get the vaccine, so I'm alienated by my company. | ワクチンを打たないので、会社で阻外されています。 |
|  | I have had allergies from vaccination before, is it okay to get the vaccine? | 以前にワクチン注射でアレルギーが出たことがあるのですが、ワクチンを打っても大丈夫ですか？ |
|  | If the PCR test is negative, is it okay? | PCR検査で陰性であれば、大丈夫ですか？ |
|  | Is it okay to drink alcohol? | お酒は飲んでも良いのですか？ |
|  | Is it true that there are rumours that vaccination will prevent you from becoming pregnant? | ワクチンをすれば妊娠しなくなるという噂がありますが本当ですか？ |
|  | Is it true that we do not have vaccines for Δ strains? | Δ株にワクチンは聞かないというのは本当ですか？ |
|  | Is karaoke dangerous? | カラオケは危険ですか？ |
|  | Is this epidemic settled next year? | この流行は来年は解決していますか？ |
|  | What is social immunity? | 社会免疫とはどのようなことですか？ |
|  | What is the cause of this virus? | このウイルスの発生原因は何ですか？ |
|  | Will I get infected if I touch something that the infected person touches? | 感染者が触れたものを触れば感染しますか？ |
| **French** | Are there neurological complications of COVID? | Complications neurologiques? |
|  | Can COVID be transmitted by kissing? | COVID transmis par un baiser? |
|  | Can I get COVID from food? | Je peux attraper COVID dans les aliments? |
|  | Can I get delta after being in remission from alpha? | Je peux avoir être infecté par delta après alpha? |
|  | Can I get infected through aerated steam? | Infection par système aeration? |
|  | Can I get reinfection? | Reinfection COVID? |
|  | Can I get vaccinated if I am on steroids? | Vaccine si cortisone? |
|  | Can my dog have COVID? | Mon chien COVID? |
|  | Do I need the third jab of vaccine? | Troisième rappel vaccin? |
|  | Is COVID dangerous in children? | COVID dangereux pour enfants? |
|  | Is delta less dangerous? | Delta moins dangeureux? |
|  | Is Pfizer better than Moderna? | Pfizer plus efficace que moderna? |
|  | Is Sinovac a good vaccine? | Sinovac bon vaccin? |
|  | Is the vaccine dangerous? | Le vaccin est il dangereux? |
|  | Is there increased risk of COVID on airplanes? | Risque accru de COVID dans l avion? |
|  | What are the current treatments against COVID? | Traitements actuel du COVID? |
|  | What is a contraindication to vaccination? | Contraindications vaccin? |
|  | What is long COVID? | Que est ce que c est le long COVID? |
|  | What is the mortality of COVID? | Mortalité du COVID? |
|  | Which is the best vaccine? | Que est le meilleur vaccin? |
| **Spanish** | Are face masks useful? | ¿Son utiles las mascarillas? |
|  | Can I serve my SHN at home? | Puedo servir a mi SHN en casa? |
|  | Does it affect the elderly more severely? | ¿Afecta de forma mas grave a los ancianos? |
|  | Does it affect to pregnant women? | ¿Afecta a mujeres embarazadas? |
|  | Does the contagion rate decrease during the summertime? | ¿La tasa de contagios disminuye durante el verano? |
|  | Does vaccines protect against the infection? | ¿Protegen las vacunas de la infección? |
|  | How fast are rapid antigen tests? | ¿Qué tan rápido son las pruebas rápidas de antígeno? |
|  | How is COVID transmitted? | Como se transmite el COVID? |
|  | How is the virus transmitted? | ¿Cómo se contagia el virus? |
|  | How many cases are there today? | ¿Cuántos casos hay hoy? |
|  | How many COVID cases are currently hospitalized? | Cuantos casos de COVID hay actualmente hospitalizados? |
|  | Can I have a positive test after being 14 days in isolated quarantine? | Puedo tener la prueba positiva despues de estar 14 dias en cuarentena aislada? |
|  | If you have passed the COVID infection, are you immune to the Coronavirus? | ¿Si has pasado la enfermedad de COVID, eres inmune al Coronavirus? |
|  | In pregnant women, does it affect the baby? | En mujeres embarazadas, afecta al bebe? |
|  | Is COVID less infective for children? | ¿Es menos transmisible en niños? |
|  | Is it frequent to need admission in intensive care units? | ¿Es frecuente necesitar ser ingresado en las unidades de cuidados intensivos? |
|  | Is it less frequent to need admission in the intensive care units in vaccinated individuals? | ¿Es menos frecuente necesitar ser ingresado en las unidades de cuidados intensivos en individuos vacunados? |
|  | Is it possible to eradicate the coronavirus? | ¿Es posible erradicar el coronavirus? |
|  | Is it transmittable via breastfeeding? | ¿Se puede contagiar por la lactancia? |
|  | Is the disease fatal? | ¿La enfermedad es mortal? |
|  | Is there an effective treatment against the virus? | ¿Existe un tratamiento efectivo contra el virus? |
|  | Is vomiting part of the symptoms of COVID? | Tener vomito es parte de los sintomas de COVID? |
|  | My parents usually come to help me with taking care of my children on weekdays. Can I still do it? | Mis padres suelen venir para ayudarme con cuidar a mis hijos los días de semana? ¿Todavía puedo hacerlo |
|  | What are coronaviruses? | ¿Qué son los coronavirus?' |
|  | What are differences with the flu virus? | ¿Cuáles son las diferencias con el virus de la gripe? |
|  | What are the main symptoms? | ¿Cuales son los principales sintomas? |
|  | What are the most frequent sequalae? | ¿Cuáles son las principales secuelas? |
|  | What are the other precautions taken at the borders? | ¿Cuáles son las otras precauciones tomadas en las fronteras? |
|  | What are the severe complications of COVID-19? | ¿Cuáles son las complicaciones severas de COVID-19? |
|  | What does COVID come from? | ¿Cual es su origen? |
|  | What if I suspect that I have been in the same place as the confirmed cases? What do I have to do? | ¿Qué pasa si sospecho que he estado en el mismo lugar que los casos confirmados? ¿Qué tengo que hacer? |
|  | What is the treatment for COVID-19? | ¿Cuál es el tratamiento para COVID-19? |
|  | What should I do if I have a side effect from the vaccine? | '¿Qué debo hacer si sufro de algún efecto secundario de la vacuna? |
|  | What should I do when I am being quarantined? How will it affect my family members? | ¿Qué debo hacer cuando estoy siendo en cuarentena? ¿Cómo afectará a mis familiares? |
|  | What should I do when I am on a leave of absence (LOA)? How will this affect my household members? | ¿Qué debo hacer cuando estoy en un permiso de ausencia (LOA)? ¿Cómo afectará esto a mis miembros del hogar? |
|  | What should I do if I had contact with someone with COVID? | ¿Que debo hacer si tuve contacto con alguien con COVID? |
|  | What vaccines are available? | ¿Qué vacunas están disponibles? |
|  | When can I have a third dose of vaccine? | Cuando puedo tener una tercera dosis de vacuna? |
|  | Why does COVID affect humans? | ¿Por qué afecta a los humanos? |
|  | Why is the virus so contagious? | ¿Por qué el virus es tan contagioso? |
| **Portuguese** | Can children have severe COVID? | Crianças podem ter COVID severa> |
|  | Can I have a positive test after 14 days in quarantine? | Posso ter um teste positivo após 14 dias em quarentena? |
|  | Do I have COVID-19 If I have a sore throat? | Eu tenho COVID-19 Se eu tiver dor de garganta? |
|  | How can I know if I have COVID? | Como posso saber se tenho COVID? |
|  | How frequently should I do ART test? | Om que frequência devo fazer ART? |
|  | How long is the incubation period? If someone travels to cities with confirmed cases, how many days without symptoms after the trip will they be considered clean and disease free? | 'Quanto tempo é o período de incubação? Se alguém viajar para as cidades com casos confirmados, quantos dias sem sintomas após a viagem, ele será considerado limpo e livre de doenças? |
|  | How quickly should I go to the doctor if I have a cough? | Com que rapidez devo ir ao médico se estiver com tosse? |
|  | I can't find a suitable mask for my son. What should I do? | Não consigo encontrar uma máscara adequada para o meu filho. O que devo fazer? |
|  | I have sore throat, can it be COVID? | Enho dor de garganta? |
|  | I received a medical license from the PHPC doctor. Can I still go out and run my errands? | Eu recebi licença médica pelo médico do PHPC. Eu ainda posso sair para correr meus recados? |
|  | I want to find out if I had COVID-19 in the past, what test could I take? | Eu quero descobrir se eu tinha COVID-19 no passado, que teste eu poderia tomar?' |
|  | I would like to seek medical attention as I have a cough and a runny nose and have recently travelled to countries with confirmed cases of COVID-19. Should I go to GP or do I have to go to A&E? | Eu gostaria de procurar atendimento médico como eu tenho uma tosse e nariz escorrendo e recentemente viajei para os países com casos confirmados de COVID-19. Devo ir para o GP ou eu tenho que ir para a A & E? |
|  | if I'm not asymptomatic, how often should I get tested? | Se eu não sou assintomático, com que frequência devo fazer o teste? |
|  | My regular GP clinic is not under the PHPC scheme. If I have flu-like symptoms, should I continue to see my regular GP or go to a different clinic that is under the PHPC regimen? | Minha clínica GP regular não está sob o esquema PHPC. Se eu tiver sintomas semelhantes à gripe, devo continuar a ver meu GP regular ou ir para uma clínica diferente que está sob o esquema PHPC? |
|  | Should I go to work if I don't feel well? | Devo ir trabalhar se não me sentir bem? |
|  | Should my baby also wear a mask? | Meu bebê também usa uma máscara? |
|  | What are the vaccine side effects? | Efeitos colaterais da vacina? |
|  | What is the difference between people who are asymptomatic or pre-symptomatic? | 'Qual é a diferença entre pessoas que são assintomáticas ou pré-sintomáticas? |
|  | Where can I take a COVID test? | Onde posso fazer um teste COVID 19? |
|  | Why are my muscles hurting? | Por que meus músculos estão doendo?'. |
